# Screening the MMV Pathogen Box reveals the mitochondrial *bc*_1_-complex as a drug target in mature *Toxoplasma gondii* bradyzoites

**DOI:** 10.7554/eLife.102511

**Published:** 2026-09-17

**Authors:** Deborah Maus, Elyzana Putrianti, Tobias Hoffmann, Michael Laue, Frank Seeber, Martin Blume

**Affiliations:** 1 P 6: Metabolism of Microbial Pathogens, Robert Koch Institute Berlin Germany; 2 ZBS 4: Advanced Light and Electron Microscopy, Centre for Biological Threats and Special Pathogens 4, Robert Koch-Institute Berlin Germany; 3 FG 16: Mycotic and Parasitic Agents and Mycobacteria, Robert Koch Institute Berlin Germany; University of the Witwatersrand South Africa; University of the Witwatersrand South Africa

**Keywords:** *Toxoplasma gondii*, compound screen, metabolomics, bradyzoite, chronic infection, energy metabolism, Other

## Abstract

The apicomplexan parasite *Toxoplasma gondii* infects 25–30% of the global human population and can cause life-threatening diseases in immunocompromised patients. The chronically infectious forms of the parasite, bradyzoites, persist within cysts in brain and muscle tissue, and are responsible for its transmission and remission of the disease. Currently available treatment options are very limited and are only effective against the fast-replicating tachyzoites, but fail to eradicate the chronic stages of *T. gondii*. The cause of these treatment failures remains unclear. Here, we utilized our recently developed human myotube-based culture model to screen compounds from the MMV Pathogen Box against pan-resistant in vitro bradyzoites, and identified multiple compounds with simultaneous activity against tachyzoites and bradyzoites. Stable isotope-resolved metabolic profiling on tachyzoites and bradyzoites identified the mitochondrial *bc*_1_-complex as a target of bradyzocidal compounds and defined their metabolic impacts on both parasite forms. Our data suggest that mature bradyzoites rely on mitochondrial ATP production.

## Introduction

*Toxoplasma gondii* is an ubiquitous apicomplexan parasite that infects virtually all warm-blooded animals, including 25–30% of humans (Montazeri et al., 2020). Of all food-borne pathogens, *T. gondii* causes 20% of the total disease burden in Europe (Havelaar et al., 2015). Although most acute infections caused by the fast replicating tachyzoite stage of the parasite remain asymptomatic in immunocompetent individuals, the parasite persists life-long in the form of encysted semi-dormant bradyzoites (Weiss and Dubey, 2009). Currently, no medical treatment exists to eradicate these tissue cysts with their rigid cyst wall from infected individuals. *T. gondii* is transmitted through undercooked meat products of infected livestock but also via feline-shed oocysts that can contaminate water sources and soil (Weiss and Dubey, 2009). Severe toxoplasmosis occurs after reactivation of cysts or primary infection of immunosuppressed individuals or fetuses of seronegative mothers and can lead to systemic infections, uveitis, encephalitis, as well as congenital transmission with potentially fatal outcomes (Montoya and Liesenfeld, 2004). Hence, tissue cysts of *T. gondii* constitute a long-term source for transmission and risk of acute disease. Also, other related cyst-forming coccidian parasites such as *Neospora caninum* and *Besnoitia besnoiti* pose an immense veterinary burden by causing abortion mainly in sheep and severe chronic infections in cattle and horses, respectively. Current treatment options exist only against the tachyzoite form of these parasites and include antifolates, such as pyrimethamine or trimethoprim, that inhibit the bifunctional dihydrofolate reductase-thymidylate synthase, in combination with sulfadiazine, which blocks the dihydropteroate synthase. These enzymes are implicated in DNA synthesis in both the parasite and side effects often require pre-mature termination of the treatment (Katlama et al., 1996a; Alday and Doggett, 2017). Another potent drug target in coccidians and other apicomplexan parasites such as *Plasmodium* spp. is the cytochrome *bc*_1_-complex in the mitochondrial respiratory chain, which is inhibited by a number of approved drugs, such as the naphthoquinones atovaquone (ATQ) (Meneceur et al., 2008; Nixon et al., 2013) and buparvaquone (BPQ) (Sharifiyazdi et al., 2012). However, both coenzyme Q analogs, including bioavailable nanosuspensions (Shubar et al., 2011), fail to eradicate encysted bradyzoites of *T. gondii* and *N. caninum* from the brains of infected mice after multiple weeks of treatment (Doggett et al., 2012; Müller et al., 2015; Doggett et al., 2020). Experimental inhibitors of the *bc*_1_-complex include 1-hydroxy-2-dodecyl-4(1H)quinolone (HDQ) that arrests the growth of asexual blood stages of the related malaria parasite *Plasmodium falciparum* (Vallières et al., 2012). However, in *T. gondii*, HDQ appears to inhibit multiple mitochondrial electron donors such as the alternative NAD-dehydrogenase and the dihydroorotate dehydrogenase (DHODH) in tachyzoites (Hegewald et al., 2013) . It does not affect mature bradyzoites in vitro (Christiansen et al., 2022), and HDQ derivatives do not clear *T. gondii* cysts from the brain of infected mice (Bajohr et al., 2010). In addition, endochin-like quinolones constitute a promising series of potent *bc*_1_-complex inhibitors that are active against *T. gondii* (McConnell et al., 2018) and *Plasmodium* parasites (Song et al., 2018). However, these compounds also do not fully eradicate the cysts in vivo (Doggett et al., 2012; Doggett et al., 2020). Similarly, bumped kinase inhibitors that selectively inhibit parasite calcium-dependent protein kinases in various apicomplexans fail to clear tissue cysts in vitro (Christiansen et al., 2022) and in vivo (Hulverson et al., 2019). Despite these efforts, it remains unclear whether the target proteins of these drugs are rendered dispensable for the survival of bradyzoites or whether the eradication failures have other reasons, such as poor permeability across the blood-brain barrier (bbb) and/or the cyst wall. To date, the genetic tools available to directly characterize the function of essential genes in bradyzoites are limited to promoter replacements (Smith et al., 2021), while no genetic methods exist to directly interrogate the fitness contribution of mitochondrially encoded genes such as the cytochrome *b* subunit of the cytochrome *bc*_1_-complex (Wang et al., 2016). Consequently, reverse pharmacological approaches that involve screening of inhibitors against target proteins remain difficult, and the underlying reasons for limited in vivo efficacies remain uncertain.

Here, we aim to address these shortcomings and identify essential processes in bradyzoite by a combination of a phenotypic screen and subsequent metabolomics-driven identification of the modes of actions of hit compounds. We employed a recently developed human myotube-based culture system to generate mature *T. gondii* drug-tolerant bradyzoites (Christiansen et al., 2022; Maus et al., 2024) to screen the MMV Pathogen Box. These in vitro cysts exhibit resistance against antifolates, bumped kinase inhibitors, and HDQ. They also express hallmarks of in vivo cysts, including oral infectivity and a cyst wall (Christiansen et al., 2022). The Medicines for Malaria Venture (MMV) Pathogen Box provides a curated set of bioactive molecules with activity against a number of pathogens, including *P. falciparum*, *Cryptosporidium parvum*, *Schistosoma* spp., and diseases caused by kinetoplastids (Veale, 2019). Untargeted and stable isotope-resolved metabolomic analyses of active drugs identified established and unsuspected modes of action in tachyzoites and bradyzoites, respectively. Our data directly show metabolic consequences of *bc*_1_-complex inhibition in bradyzoites and suggest its crucial role in ATP production.

## Results

### The Pathogen Box contains dually active compounds

**Figure 1.**
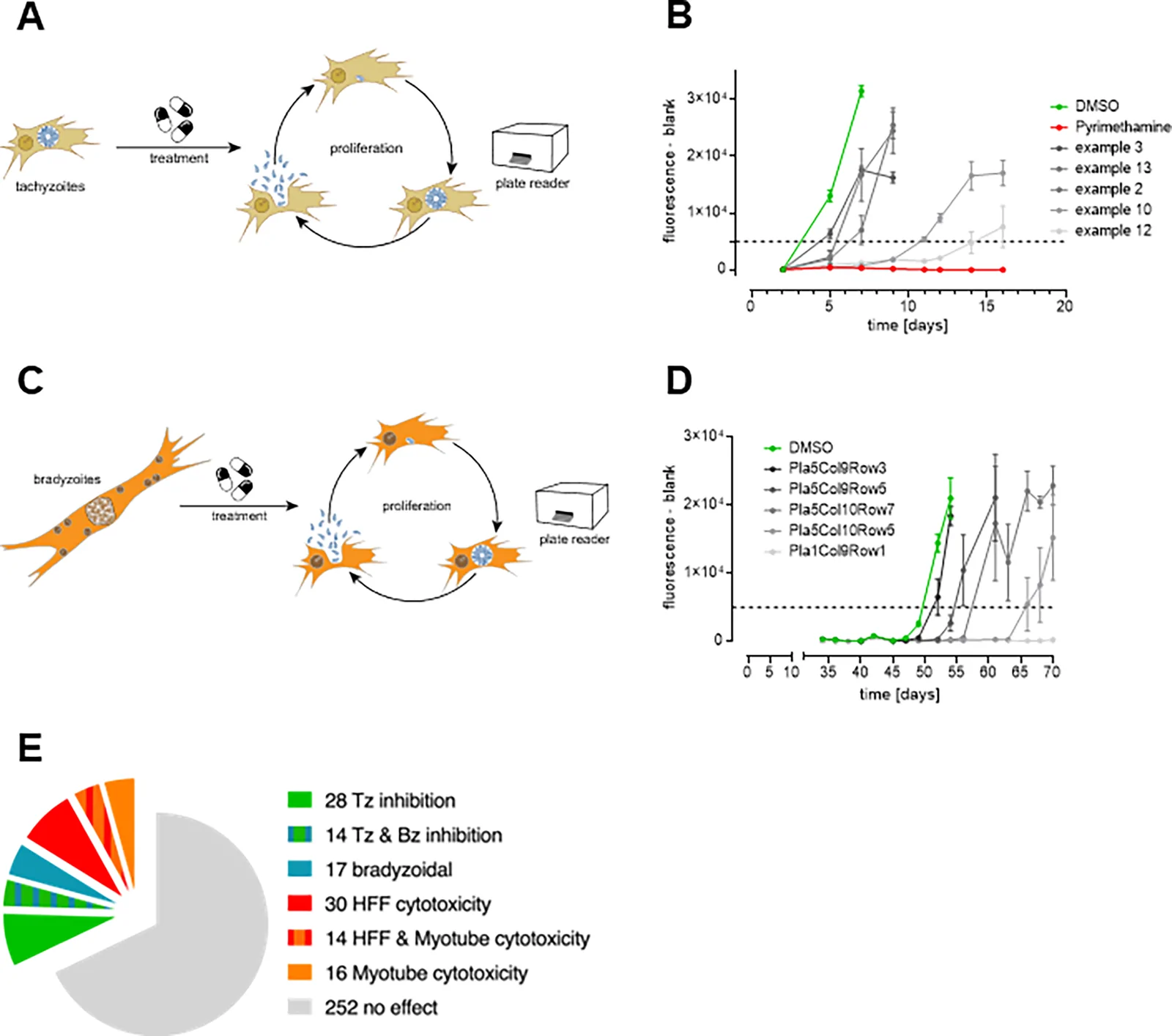
An in vitro screen reveals tachy- and bradyzocidal properties of the Medicines for Malaria Venture (MMV) Pathogen Box compounds. (**A**) Experimental scheme of the MMV compound screen on *T. gondii* tachyzoites in human foreskin fibroblast cells. (**B**) Growth curves of tachyzoites treated with 10 µM of each MMV compound for 7 days (shades of gray), a solvent control (green), and an inhibition control (red). (**C**) Experimental scheme of the MMV compound screen on bradyzoites matured in myotubes. (**D**) Growth curves of bradyzoites treated with 10 µM of each compound (shades of gray) or a solvent control (green) for 7 days, followed by a tachyzoite regrowth phase. (**E**) Pie chart displaying the number of Pathogen Box compounds and their effects on our in vitro cultures.

To initially identify compounds that simultaneously target the tachyzoite and the bradyzoite stage of *T. gondii*, we tested 371 of 400 compounds from the Pathogen Box at a concentration of 10 µM against both stages. We excluded previously reported active compounds against *T. gondii* tachyzoites (Spalenka et al., 2018) from this initial screen. Human foreskin fibroblast (HFF) cells were infected with tachyzoites of the Pru strain expressing tandem Tomato fluorescent protein in its cytosol (PruTom) at a multiplicity of infection (MOI) of 1 (Figure 1A). 47 compounds completely inhibited growth during 7 days of exposure, while the parasitemia of DMSO-treated controls already peaked after 4 days (Figure 1B). To also identify bradyzocidal compounds, we differentiated KD3 myoblasts for 1 week into multinucleated myotubes and infected them under bicarbonate-deplete conditions and at an MOI of 0.1 for 4 weeks to allow maturation of tissue cysts (Figure 1—figure supplement 1). We exposed the cultures to 10 µM of the 371 MMV compounds for 1 week, removed the inhibitors, and stimulated re-differentiation into tachyzoites by bicarbonate supplementation (Figure 1C). Growth of DMSO-treated cultures became detectable after 10–14 days. Interestingly, 31 compounds prevented any tachyzoite regrowth (Figure 1D), indicating their bradyzocidal activity. To exclude host cytotoxicity-dependent suppression of parasite growth, we measured cell viability via the reductive potential of myotubes and HFF cells using a resazurin assay. At 10 µM, 30, 12, and 13 compounds inflicted cytotoxicity onto HFFs, myotubes, or both cell types, respectively (Supplementary file 1). This initial screen identified 14 compounds with activity against both tachyzoites and bradyzoites and no apparent cytotoxicity (Figure 1E).

**Figure 1—figure supplement 1.**
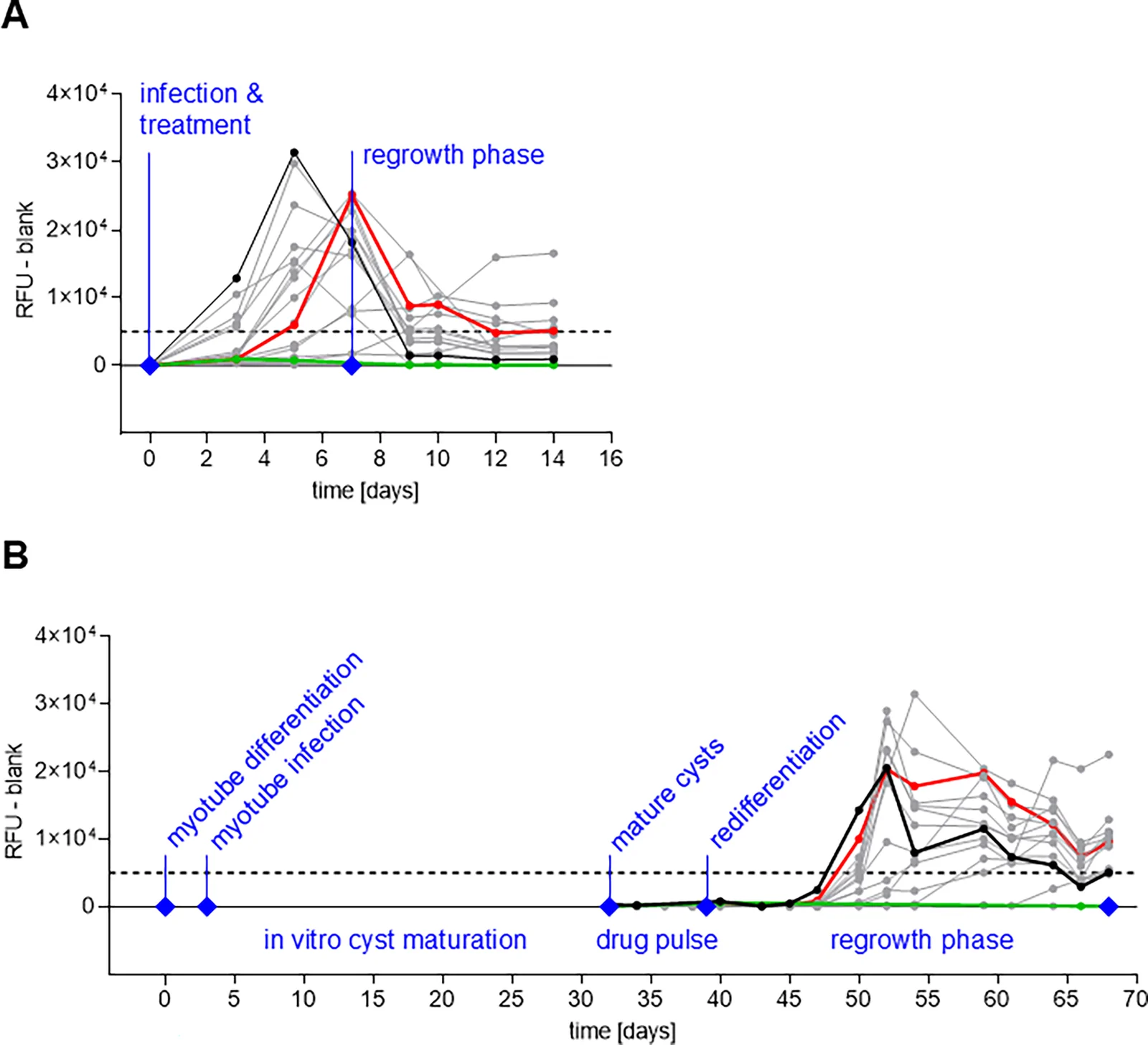
Longitudinal screening procedure. All 400 compounds of the Medicines for Malaria Venture (MMV) Pathogen Box were screened against (**A**) tachyzoites (n=4) and (**B**) in vitro tissue cysts (n=3). Plotted fluorescence values were background-subtracted and reflect the abundance of parasites per treatment over time (black line = solvent control, green line = inhibitory compound, red line = ineffective compound, dashed line = growth threshold).

### Validation of screening hits

**Figure 2.**
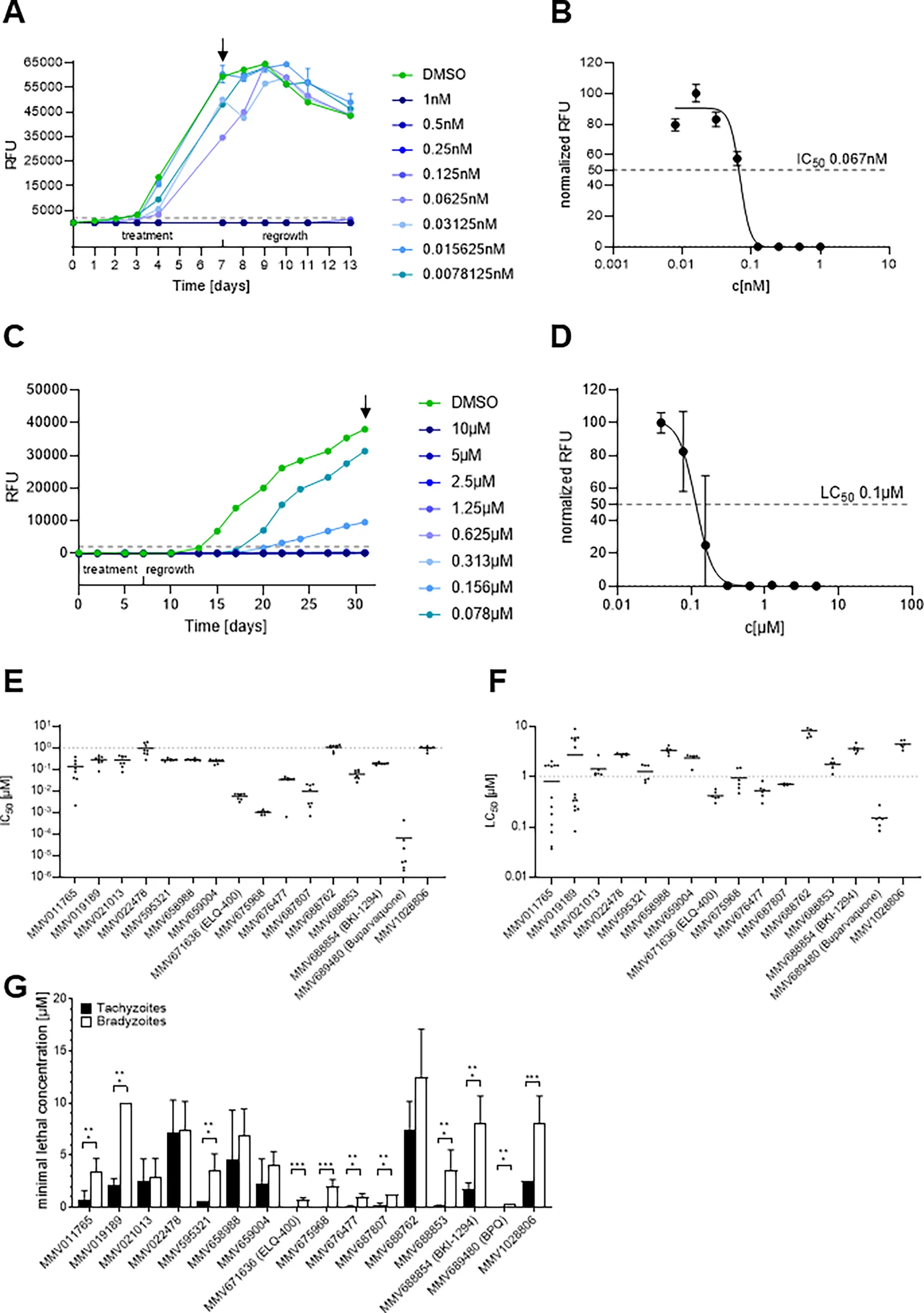
Confirmed Pathogen Box compounds that target both tachyzoites and mature bradyzoites. (**A**) shows the growth curve of MMV689480 (buparvaquone), one of the 16 dually active compounds, in a tachyzoite growth assay (n=8). The infected cultures were treated for 7 days, and regrowth in absence of the inhibitor was observed for another week. Depending on the development of the DMSO control, the half-maximal inhibitory concentration (IC_50_) was determined at either day 4 or 7 (arrow). (**B**) Shown is the respective data plotted as relative fluorescence units depending on the concentration at day 7. (**C**) With 4 weeks of age, mature bradyzoite cultures (n=6), the half-maximal lethal concentration (LC_50_) was determined at the time point which ideally reflected the dose-response curve (in the case of buparvaquone at day 31; arrow). (**D**) The LC_50_ of buparvaquone on encysted bradyzoites determined at day 31. All IC_50_ and LC_50_ of dually active compounds are summarized in (**E**) for tachyzoite and (**F**) for bradyzoite cultures. The gray dotted line indicates 1 µM in both charts. (**G**) Comparison of the minimal lethal concentration of each compound on tachyzoites and bradyzoites. Mann-Whitney test of eight replicative measurements on tachyzoites and six replicates of bradyzoites from two independent experiments each ***p<0.001.

To validate our results, we determined half-inhibitory concentrations (IC_50_) in PruTom tachyzoites (Figure 2A and B) and half-maximal lethal concentration (LC_50_) in bradyzoites. We also included 29 compounds that were previously reported to target tachyzoites (Spalenka et al., 2018). We confirmed dual activity of 16 compounds that exhibited mean IC_50_ values ranging between 70 pM and 1.1 µM for tachyzoites (Figure 2E—figure supplement 1) and LC_50_ values between 0.15 and 8.2 µM for bradyzoites (Figure 2F—figure supplement 1D). To allow a comparison between the tachyzoite and bradyzoite data, we also determined the respective minimal lethal concentrations (MLCs) over a 7-day treatment duration of tachyzoites as well (Figure 2G). Except for four compounds that were not significantly different (MMV021013, MMV022478, MMV658988, MMV65900), MLCs appear generally higher in bradyzoites, potentially reflecting their lower metabolic activity and/or the cyst wall as a diffusion barrier.

**Figure 2—figure supplement 1.**
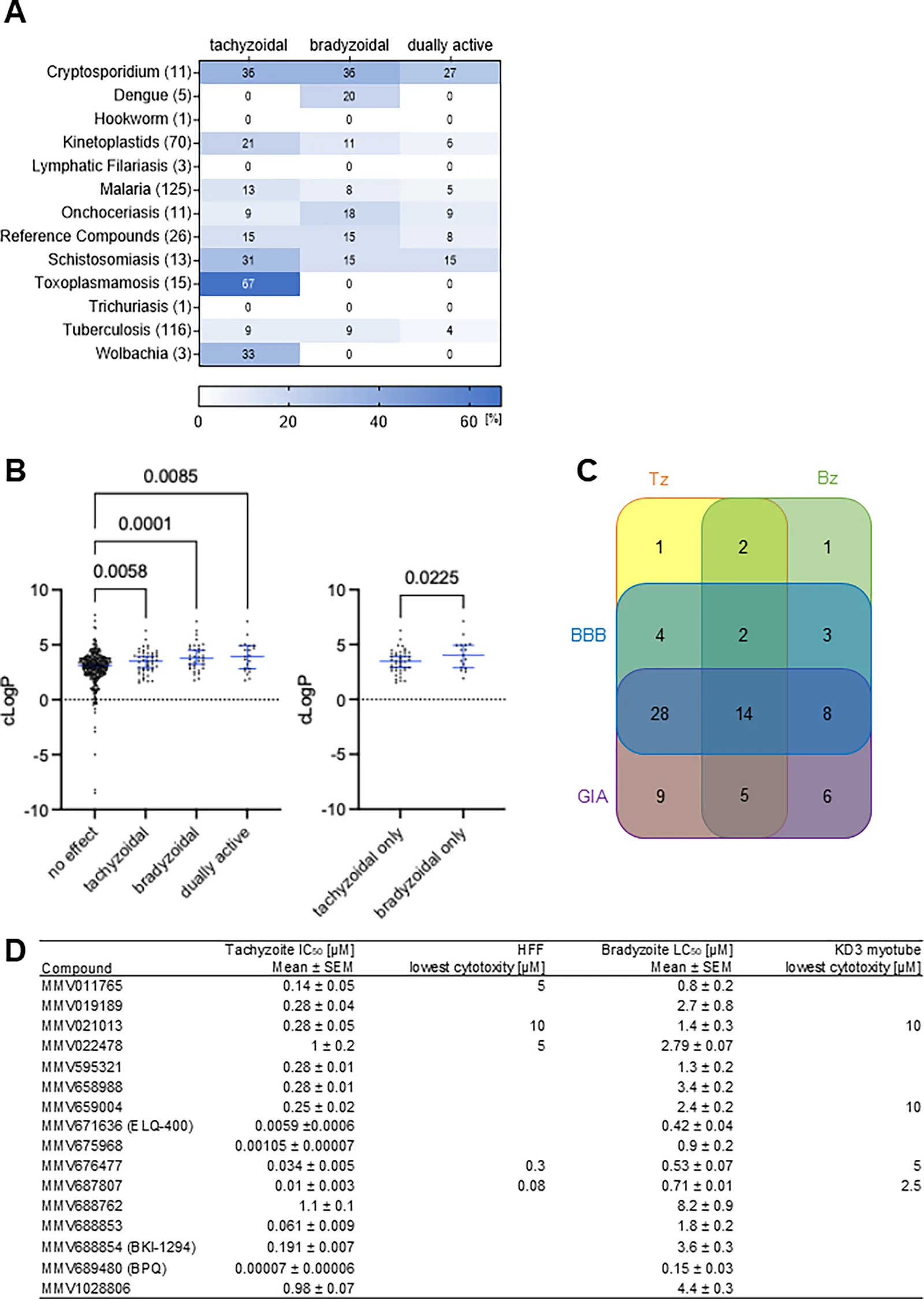
Properties of dually active screening hits. (**A**) Table showing the relative amount of tachyzocidal, bradyzocidal, and dually active compounds per originally indicated target species. For example, 11 compounds were indicated by Medicines for Malaria Venture (MMV) to be active against *Cryptosporidium,* and 36% and 27% of these are bradyzocidal and dually active, respectively. In contrast, 67% of the 15 compounds indicated as active against *Toxoplasma* possessed activity against tachyzoites, but none were active against bradyzoites. (**B**) Estimated lipophilicity approximated by predicted partition coefficient suggests that active compounds generally exhibit an increased lipophilicity compared to inactive compounds. (**C**) A Venn diagram showing intersections of tachyzocidal and bradyzocidal compounds as well as their predicted gastrointestinal absorption capability (GIA) and blood-brain barrier (bbb) permeability. (**D**) Tabulation of compound IC_50_ and LC_50_ data of confirmed active compounds. The calculated IC_50_s and LC_50_s of 16 dually active compounds from Figure 2 are summarized in this table. The tachyzoite IC_50_ values represent eight replicates, the LC_50_ values were generated from six replicate bradyzoite cultures. Lowest cytotoxicity values were recorded during the primary screen as detailed in the Materials and methods section.

### Dually active MMV Pathogen Box hits share high lipophilicity and are indicated to be active against other pathogens

To gain insight into general properties of active compounds from our initial screen, we tabulated indicated activities as provided by MMV (Figure 2—figure supplement 1). As expected, these compounds were mostly earmarked as active against *T. gondii* tachyzoites (67%); however, none of those compounds exhibited bradyzocidal activity. Instead, 27% of dually active compounds were considered active against *Cryptosporidium*. Also, despite the relative phylogenetic proximity of *P. falciparum*, only 5% of the 125 potential antimalarials blocked growth of both *T. gondii* stages. To characterize physicochemical properties of these dually active hits, we approximated their lipophilicity by their logP values (Supplementary file 1) and compared them to compounds that were inactive against *T. gondii* (Figure 2—figure supplement 1). We found that tachyzocidal, bradyzocidal, and dually active compounds possess a statistically significantly higher lipophilicity than non-active compounds, and this trend appeared more accentuated for bradyzocidal and dually active compounds. However, when comparing tachyzocidal with bradyzocidal compounds, including dually active ones, we did not find a difference (p=0.06). We estimated permeability through the bbb using LightBBB (Shaker et al., 2021) and gastrointestinal absorbability using SwissADME (Daina et al., 2017; Supplementary file 1). Most of the dually active drugs were predicted to be both permeable across the bbb and absorbed in the intestine (Figure 2—figure supplement 1). Together, these data support phylogenetic relationships as a weak predictor of the activity of Pathogen Box compounds against *T. gondii* bradyzoites and highlight the role of lipophilicity among the compounds tested here.

### Metabolic drug responses in tachyzoites indicate conserved and novel modes of action

**Figure 3.**
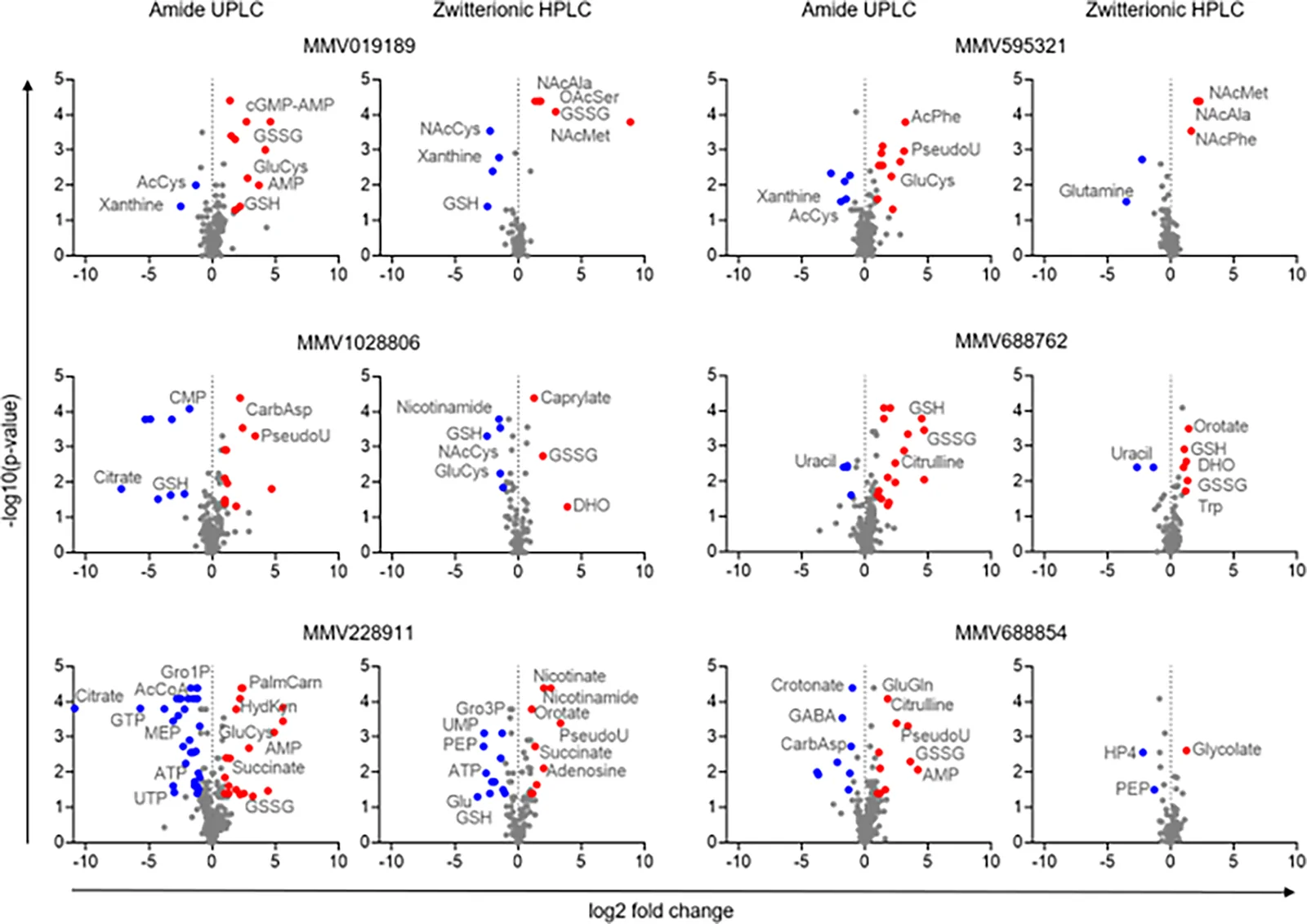
Untargeted metabolomics of tachyzoites treated with six screening hits effective against both stages of *T*. *gondii*. Volcano plots showing the parasitic, intracellular metabolic phenotype caused by Medicines for Malaria Venture (MMV) Pathogen Box compounds that inhibited tachyzoites and bradyzoites during the screen. The metabolome was measured with two columns of complementary chemistries to enhance metabolite coverage. Blue dots indicate metabolites that were significantly reduced compared to DMSO-treated parasites, and red dots indicate significantly accumulated metabolites (p<0.05, log2 fold change <-1 or >1, 3× biological replicates with 3× technical replicates each, n=9). Abbreviations: AcCoA, acetyl-CoA; AcCys, acetylcysteine; AcPhe, acetylphenylalanine; AMP, adenosine monophosphate; ATP, adenosine triphosphate; CarbAsp, carbamoyl aspartate; cGMP-AMP, cyclic guanosine monophosphate-adenosine monophosphate; CMP, cytosine monophosphate; DHO, dihydroorotate; GABA, gamma-aminobutyric acid; GluCys, glutamylcysteine; GluGln, glutamylglutamine; Gro1P, glyceraldehyde 1-phosphate; Gro3P, glyceraldehyde 3-phosphate; GSH, glutathione oxidized; GSSG, glutathione reduced; GTP, guanosine triphosphate; HP4, hexose phosphate No. 4 (fourth chromatographic peak of 260.02972); HydKyn, hydroxylkynurenine; MEP, 2-C-methyl-D-erythritol 4-phosphate; NAcAla, *N*-acetylalanine; NAcCys, *N*-acetylcysteine; NAcMet, *N*-acetylmethionine; NAcPhe, *N*-acetylphenylalanine; OAcSer, *O*-acetylserine; PalmCarn, palmitoylcarnitine; PEP, phosphoenolpyruvate; PseudoU, pseudouridine; Trp, tryptophan; UMP, uridine monophosphate; UTP, uridine triphosphate.

We chose to focus on dually active compounds because they are clinically more relevant, and investigation of their targets allows comparisons between parasite stages. To that end, we chose five compounds based on their availability and yet unknown mode of action (Figure 3—figure supplement 1, Supplementary file 2) to test metabolite responses of tachyzoites. Infected monolayers were treated for 3 hr with compound concentrations five times their respective IC_50_ values or the solvent DMSO. The inhibitor MMV688854 (BKI 1294) (Doggett et al., 2014) is considered to inhibit a non-metabolic target (calcium-dependent protein kinase [CDPK1]) at the employed concentration (Hayward et al., 2023) and served as an additional control to estimate the metabolic impact of growth inhibition. We filter-purified parasites from quenched monolayers and analyzed their acetonitrile extracts by LCMS using both pHILIC and BEH-amide chromatography columns. We generated nine samples per condition from three repeats of the experiment with triplicate samples. A principal component analysis illustrates that batch effects are the main contributors to biological variances within the dataset (Figure 3—figure supplement 1). Despite that, all compounds elicited a distinct metabolic phenotype on both LCMS systems (Figure 3). The CDPK1 inhibitor increased levels of AMP, oxidized glutathione, pseudo uridine, citrulline, glycolate, and glutamyl glutamine, while reducing the abundances of GABA, carbamoyl aspartate, phosphoenolpyruvate, and a hexose phosphate. Shifts in these metabolites are shared among many treatments and indicate their implication in a general stress response and thus do not signify direct interference with parasite metabolism.

**Figure 3—figure supplement 1.**
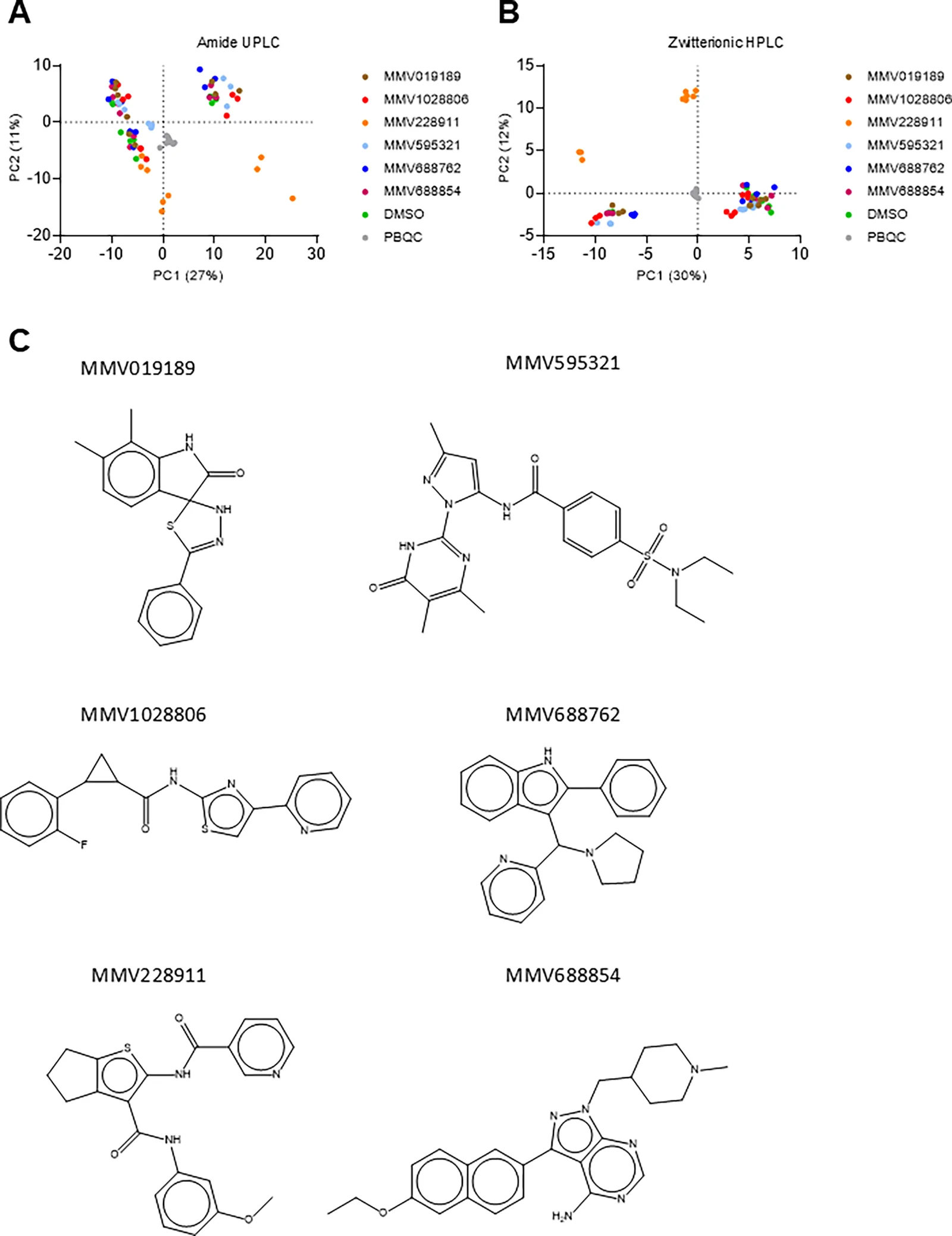
Principal component analysis of LCMS metabolomes of treated parasites using two chromatographic columns. (**A**) Principal component analysis of metabolites from extracellular tachyzoites that were treated with indicated Medicines for Malaria Venture (MMV) compounds. Metabolites were separated using BEH-amide column-HILIC chromatography and detected by mass spectrometry. (**B**) The same metabolite extracts have been re-analyzed using pHILIC-column HILIC chromatography for separation instead. (**C**) Chemical structures of dually active and tested compounds as provided by MMV.

Inhibitor-specific responses include the accumulation of cGMP-AMP after treatment with MMV019189, accumulation of carbamoyl aspartate and dihydroorotate upon MMV1028806 treatment, and a general depletion of central carbon metabolites (succinate, citrate, glyceraldehyde 3-phosphate, ATP, 2-C-methylerythritol 4-phosphate [MEP]) in MMV228911-treated parasites. MMV595321 caused an accumulation of N-acetylated amino acids, and MMV688762 led to uracil deficiency and an accumulation of dihydroorotate and orotate. These data suggest that growth inhibitors cause a bipartite metabolic response composed of generic and compound-specific changes. While these findings highlight compound-specific metabolic signatures, some observed alterations may represent downstream or systemic effects of inhibitor action, reflecting the inherent complexity of parasite metabolism.

### MMV1028806 and BPQ elicit metabolic responses similar to the *bc*_1_-complex inhibitor ATQ

**Figure 4.**
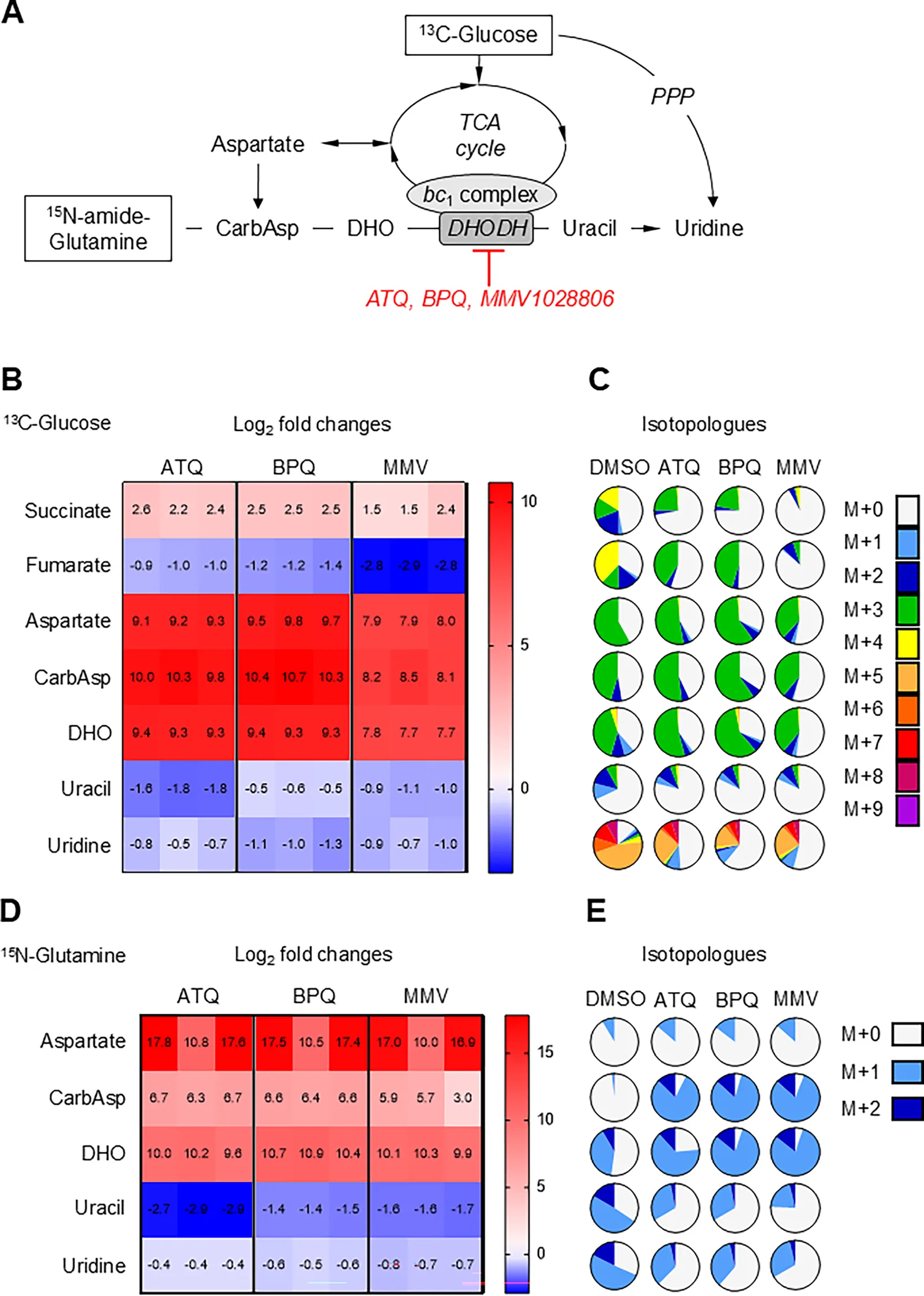
The metabolic response to electron transport chain inhibitors and MMV1028806. (**A**) Scheme of the affected pathways upon *bc*_1_-complex inhibition. Intracellular tachyzoites were grown in the presence of U-^13^C-glucose (**B, C**) or ^15^N-amide-glutamine (**D, E**) instead of unlabeled carbon sources and treated with atovaquone (ATQ), buparvaquone (BPQ), and MMV1028806 (MMV) for 3 hr. (**B, D**) The treatment-induced changes of mitochondrial electron transport chain (mETC)-related metabolite abundances are shown as log_2_-fold changes in comparison to DMSO-treated cultures. Shown are three replicate measurements. (**C, E**) Shown is the average isotopologue distribution of mETC-related metabolites. The pie charts depict the number of heavy carbon or nitrogen atoms incorporated into the respective metabolites (M+0 in light gray represents the unlabeled metabolite, while M+1 in light blue indicates the integration of one heavy atom into the molecule). Shown are the means of three replicate measurements. Abbreviations: ATQ, atovaquone; BPQ, buparvaquone; CarbAsp, carbamoyl-aspartate; DHO, dihydroorotate; DHODH, dihydroorotate dehydrogenase; GABA, gamma-aminobutyrate; MMV, MMV1028806; PPP, pentose phosphate pathway; SSA, succinic semialdehyde.

The metabolic impact of most tested MMV compounds indicated a novel mode of action that did not resemble known or recognizable metabolic fingerprints. However, the metabolic fingerprint of MMV1028806 was consistent with inhibition of pyrimidine biosynthesis at the *bc*_1_-complex-dependent oxidation reaction of dihydroorotate to orotate, as has been reported for *P. falciparum* (Figure 4A; Allman et al., 2016; Creek et al., 2016; Antonova-Koch et al., 2018). A similar response would be expected to BPQ, which we found to be dually active against *T. gondii* tachyzoites and bradyzoites and which targets the *bc*_1_-complex in *Theileria annulata* (McHardy et al., 1985), *Neospora caninum* (Müller et al., 2015), and *T. gondii* tachyzoites (Hayward et al., 2023).

**Figure 4—figure supplement 1.**
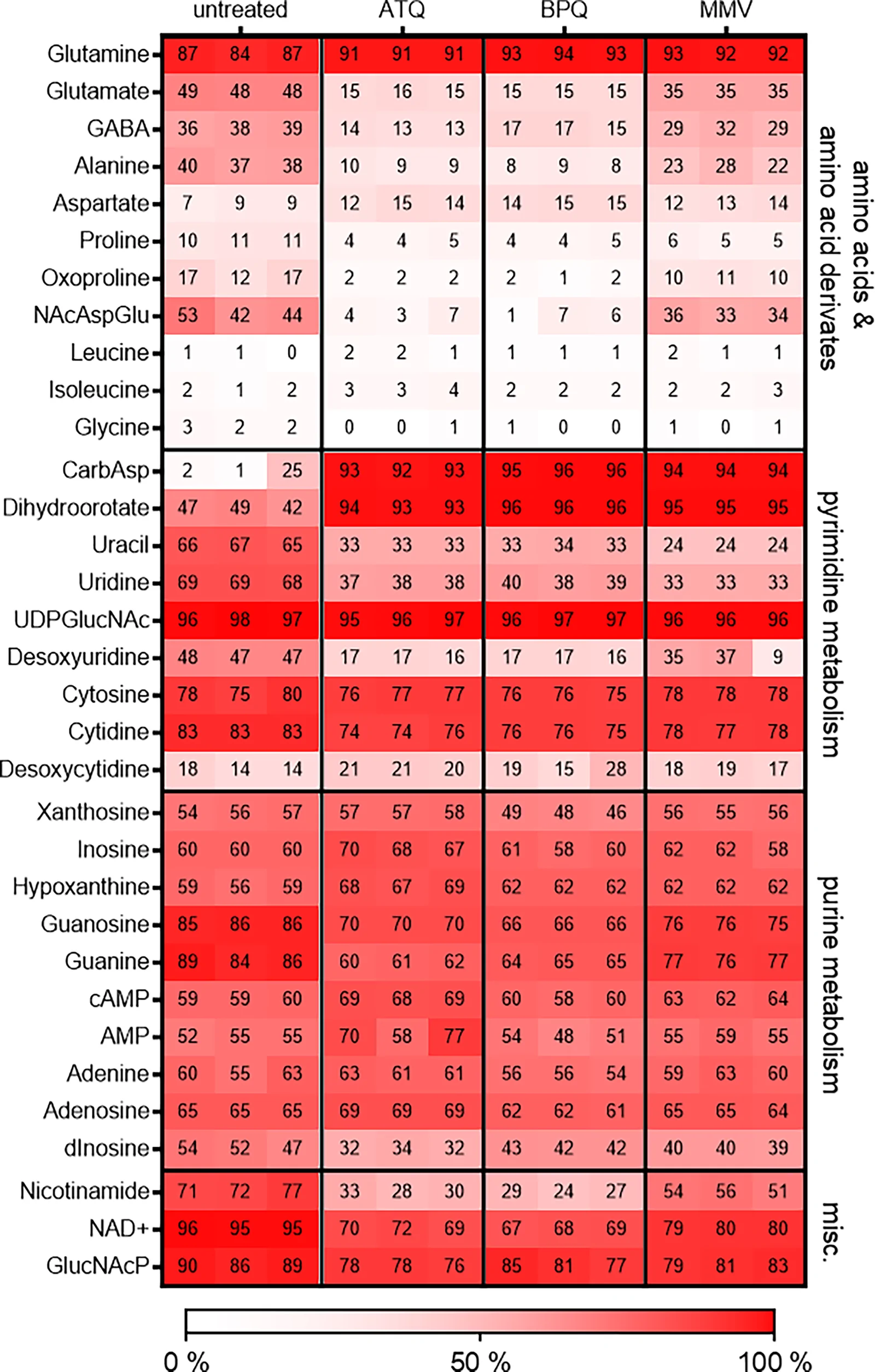
Intracellular tachyzoites exhibit differential ^15^N incorporation after treatment with mitochondrial electron transport chain (mETC) inhibitors. RH∆ku80 were treated with atovaquone, buparvaquone, and MMV1028806, labeled with ^15^N-amide-L-glutamine for 3 hr, followed by their isolation from the human foreskin fibroblast (HFF) cells. The heatmap shows the overall isotopic labeling (100-’M+0’) of each replicate.

To test these hypotheses, we exposed intracellular parasites to MMV1028806 or BPQ while substituting glucose with ^13^C6-glucose for 3 hr. We then measured the incorporation of ^13^C into the central carbon metabolites, as well as the intermediates of the pyrimidine synthesis pathway. As controls, we utilized the known *bc*_1_-complex inhibitor ATQ and the solvent DMSO (Supplementary file 3). Treatment with all three inhibitors led to a dramatic accumulation of +3 labeled aspartic acid, carbamoyl aspartate, and dihydroorotate, which was accompanied by a depletion of uracil and uridine, in particular of the +5 labeled uridine isotopologue. These changes are consistent with impaired function of the *bc*_1_-complex-dependent DHODH (Figure 4B and C). Another common effect was the accumulation of succinate and depletion of fumarate, suggesting reduced recycling of FADH_2_ via the *bc*_1_-complex.

To independently verify our results, we hypothesized that *T. gondii* also incorporates glutamine-derived nitrogen into pyrimidines. We replaced glutamine with its ^15^N-amide-labeled isotopologue in the tachyzoite culture media for 3 hr while simultaneously exposing the parasites to ATQ, BPQ, or MMV1028806. As expected from our previous experiments, we observed a marked decrease of uracil and uridine levels while aspartate, carbamoyl aspartate, and dihydroorotate accumulated again (Figure 4D, Supplementary file 4). ^15^N was incorporated into many metabolites (Figure 4—figure supplement 1). Consistently, labeling of pyrimidines was diminished while carbamoyl aspartate and dihydroorotate were heavily labeled as M+1 isotopologues (Figure 4E).

Together, these data show that treatments with all three compounds lead to the accumulation of metabolites upstream of the *bc*_1_-complex-dependent reactions during pyrimidine biosynthesis and the TCA cycle, and to the depletion of downstream metabolites (Figure 4A). These metabolic signatures suggest an inhibition of the *T. gondii bc*_1_-complex in tachyzoites.

### ATQ, BPQ, and MMV1028806 decrease the mitochondrial membrane potential of tachyzoites and bradyzoites and block the electron transport chain

**Figure 5.**
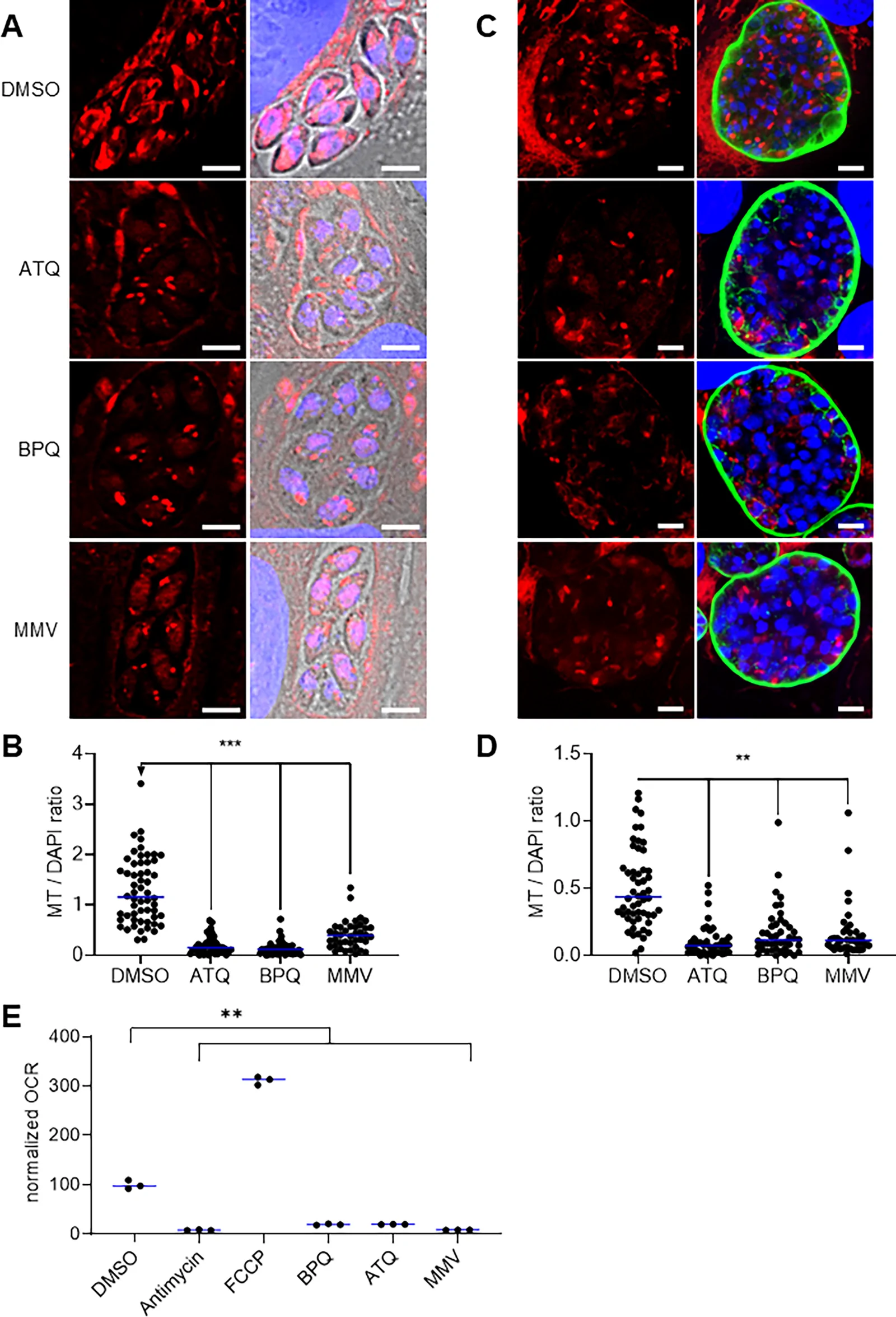
Atovaquone (ATQ), buparvaquone (BPQ), and MMV1028806 reduce mitochondrial potential and respiration in *T*. *gondii*. (**A**) Human foreskin fibroblast (HFF) cells were infected with tachyzoites and treated with DMSO, ATQ, BPQ, and MMV1028806 for 24 hr, and mitochondria were stained with MitoTracker (MT, red), and DNA was stained with DAPI (blue). Scale bar = 5 µm. (**B**) The ratios of fluorescence intensities between MT and DAPI dyes were calculated per vacuole (DMSO n=56, ATQ n=45, BPQ n=49, MMV1028806 n=40; blue lines represent the median, two-sided Mann-Whitney test). (**C**) 4 weeks of age, myotube-derived ME49 in vitro cysts were treated with DMSO, ATQ, BPQ, and MMV1028806 for 24 hr, and mitochondria were stained with MT (red), the cyst wall was stained with *Dolichos biflorus* agglutinin and Streptavidin-Cy2 (green), and DNA was stained with DAPI (blue). Scale bar = 20 µm. (**D**) The ratios of fluorescence intensities between MT and DAPI dyes were calculated per vacuole (DMSO n=56, ATQ n=42, BPQ n=45, MMV1028806 n=38; blue lines represent the median, two-sided Mann-Whitney test). (**E**) 10 million freshly egressed tachyzoites were incubated with known *bc*_1_-complex inhibitors ATQ and BPQ, specific control compounds antimycin and 4-(trifluoromethoxy)phenylhydrazone (FCCP), and MMV1028806. Every treatment significantly impacts the oxygen consumption rate (OCR) (blue line represents the median, n=3) (Welch’s test; **p<0.005).

The inhibition of the *bc*_1_-complex by ATQ leads to the collapse of the mitochondrial membrane potential in *T. gondii* tachyzoites (Vercesi et al., 1998), and we anticipated a similar phenotype in BPQ or MMV1028806-treated parasites. We treated intracellular tachyzoites and bradyzoites for 24 hr with the three inhibitors or 0.1% DMSO alone and estimated the mitochondrial membrane potential and morphology using a fluorescent MitoTracker dye. The mitochondria of control tachyzoites appeared as extended loops but largely collapsed after treatments with all three compounds (Figure 5A and B). Notably, also host mitochondria appear influenced. To evaluate the specificity of this assay, we used pyrimethamine, clindamycin, and 6-diazo-5-oxonorleucine (DON) that target dihydrofolate reductase, apicoplast translation, and glutaminase as controls. MitoTracker and GFP signal intensities remained comparable to DMSO controls (Figure 5—figure supplement 1). DAPI signal intensity appeared reduced in pyrimethamine-treated samples. In bradyzoites, mitochondria appeared mostly as spheres or were donut-shaped (Figure 5—video 1), and their relative fluorescence decreased after 24 hr treatment with ATQ, BPQ, or MMV1028806 at 5 µM, 500 nM, or 5 µM, respectively, while their morphology appeared unchanged (Figure 5C and D). Analysis by thin section electron microscopy revealed a largely unaffected sub-mitochondrial ultrastructure, but the areas of mitochondrial profiles were changed in comparison to control after exposure with ATQ and MMV1028806 but not with BPQ (Figure 5—figure supplement 2). This indicates that the latter decreases MitoTracker fluorescence intensity directly, while ATQ and MMV1028806 cause both a drop in MitoTracker fluorescence and a reduced size of the mitochondria, which leaves open whether a change of the membrane potential, of the size, or even of both parameters were responsible for the drop in MitoTracker fluorescence.

**Figure 5—figure supplement 1.**
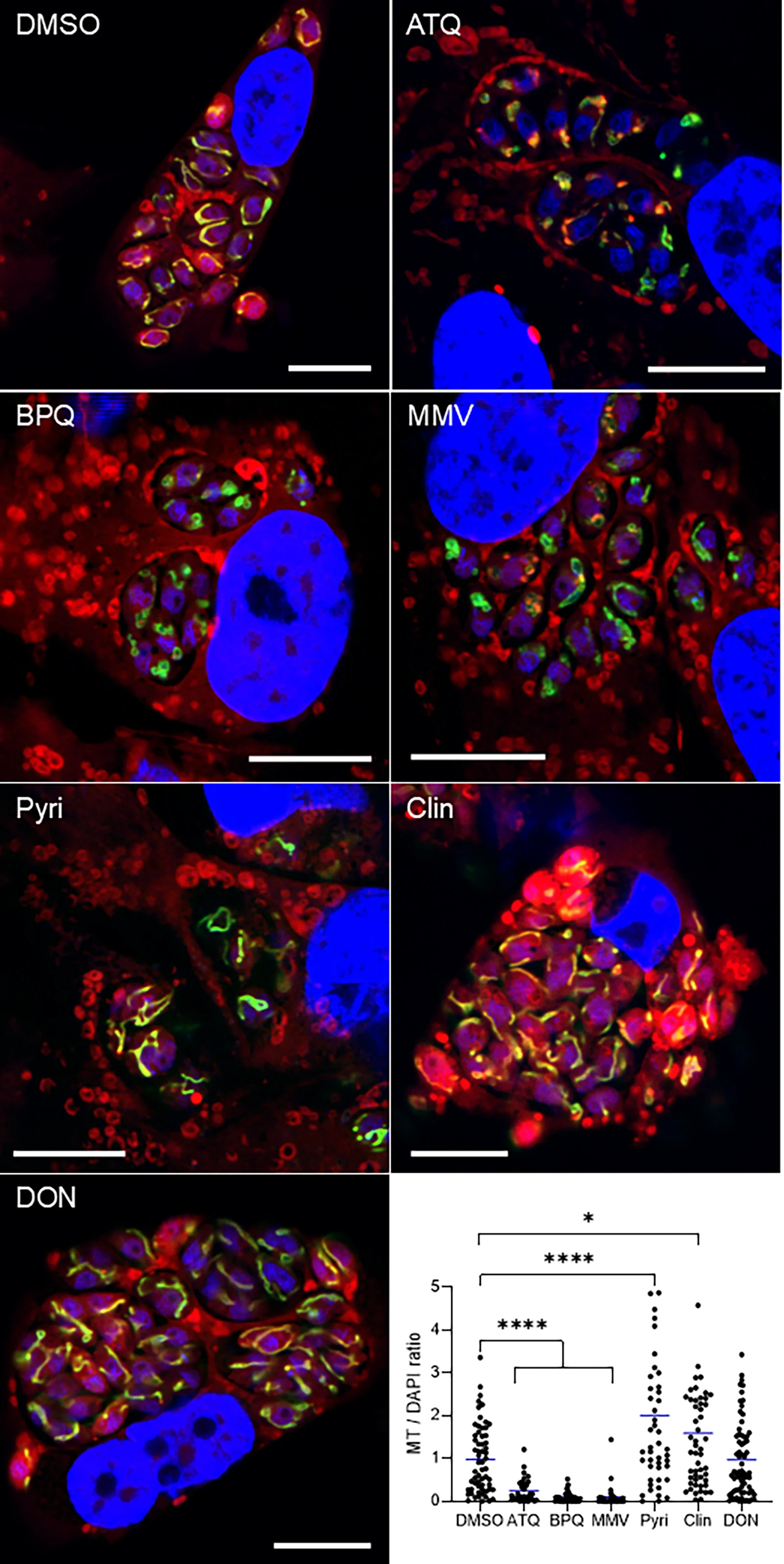
Direct mitochondrial inhibitors reduce MitoTracker (MT) signal intensity and MT/DAPI ratio in *T*. *gondii* tachyzoites. Human foreskin fibroblast (HFF) cells were infected with RH-S9 tachyzoites and treated for 24 hr with DMSO (n=61), atovaquone (ATQ, n=32), buparvaquone (BPQ, n=35), MMV1028806 (MMV, n=36), pyrimethamine (Pyri, n=58), clindamycin (Clin, n=53), and 6-diazo-5-oxonorleucine (DON, n=68). * and **** denote p<0.05 and p<0.000 in Mann-Whitney tests. MT signal appears red, DAPI stain is blue, and the mitochondrial signal originates from GFP. Scale bars represent 10 µm.

**Figure 5—figure supplement 2.**
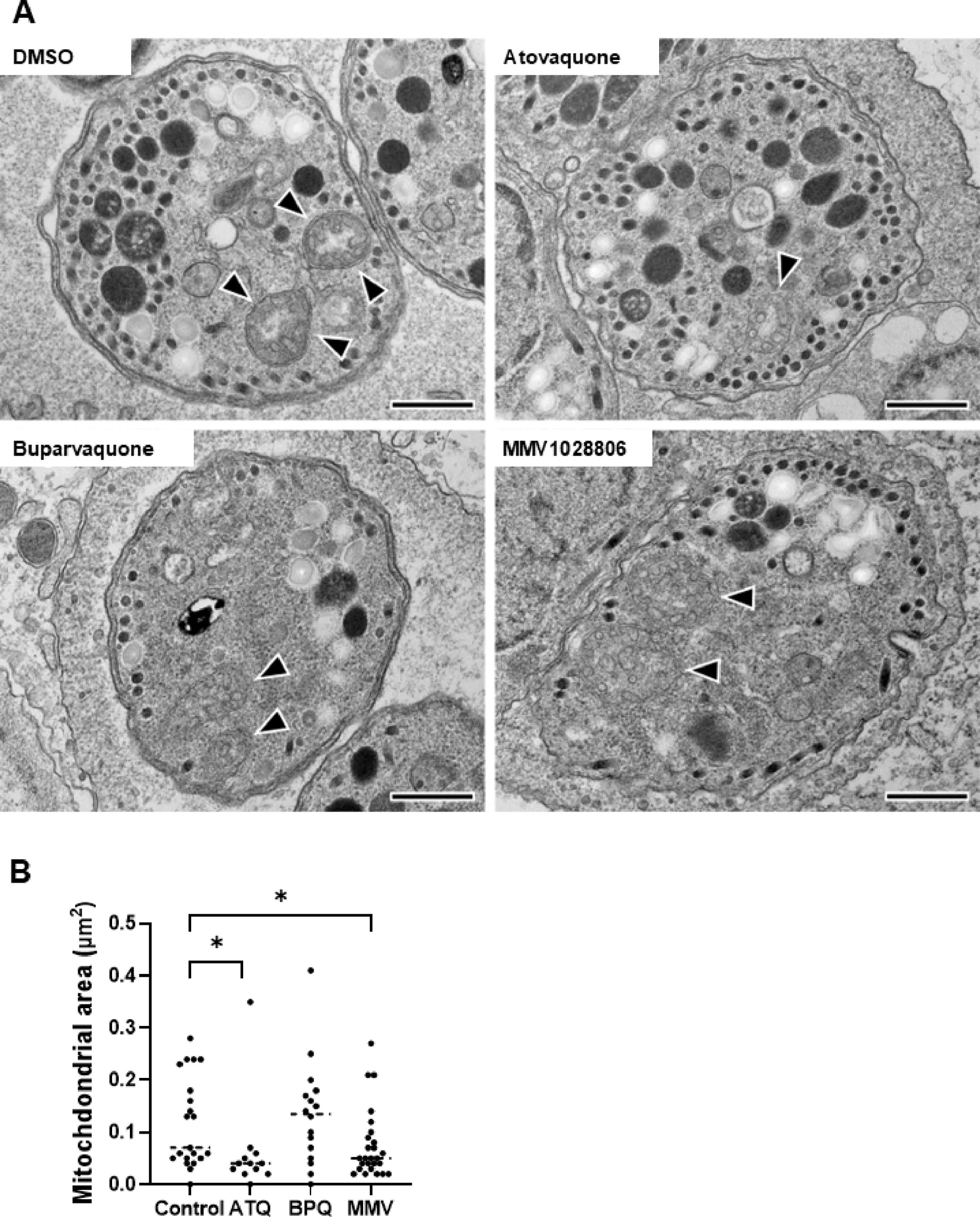
*bc*_1_-complex inhibitors partially reduce mitochondrial profiles of bradyzoites. Thin section electron microscopy of cysts that were treated with atovaquone (ATQ), buparvaquone (BPQ), or MMV1028806 (MMV) for 24 hr. Scale bar 0.5 µm. (**A**). (**B**) Measured areas of mitochondrial profiles from 21, 12, 15, and 26 images showing DMSO-, ATQ-, BPQ-, and MMV1028806-treated parasites (* denotes p<0.05 in Mann-Whitney tests).

**Figure 5—video 1.** Fluorescence microscopy of *T. gondii* tissue cyst with mitochondria. Fluorescence microscopic reconstruction of a 4-week-old tissue cyst of the ME49 strain. *Dolichos biflorus* agglutinin (DBA) staining shows the cyst wall in green, DAPI stains DNA shown in blue, and MitoTracker was used to stain mitochondria in red.

We next tested directly whether the mitochondrial electron transport chain (mETC) was blocked by ATQ, BPQ, and MMV1028806 by measuring oxygen consumption of treated tachyzoites using a fluorescence-based assay (Figure 5E). The solvent DMSO, the decoupling agent carbonyl cyanide 4-(trifluoromethoxy)phenylhydrazone (FCCP), and the *bc*_1_-complex inhibitor antimycin A served as controls. Treatment with FCCP led to a marked increase in oxygen consumption compared to the DMSO control, indicating maximal respiratory capacity of the parasites. In contrast, treatments with ATQ, BPQ, MMV1028806, and antimycin A resulted in substantially reduced oxygen consumption levels relative to the DMSO control and suggest indeed a blockage of the mETC consistent with the inhibition of the *bc*_1_-complex.

### Inhibitors of the mitochondrial electron transport chain alter catabolic processes in bradyzoites

**Figure 6.**
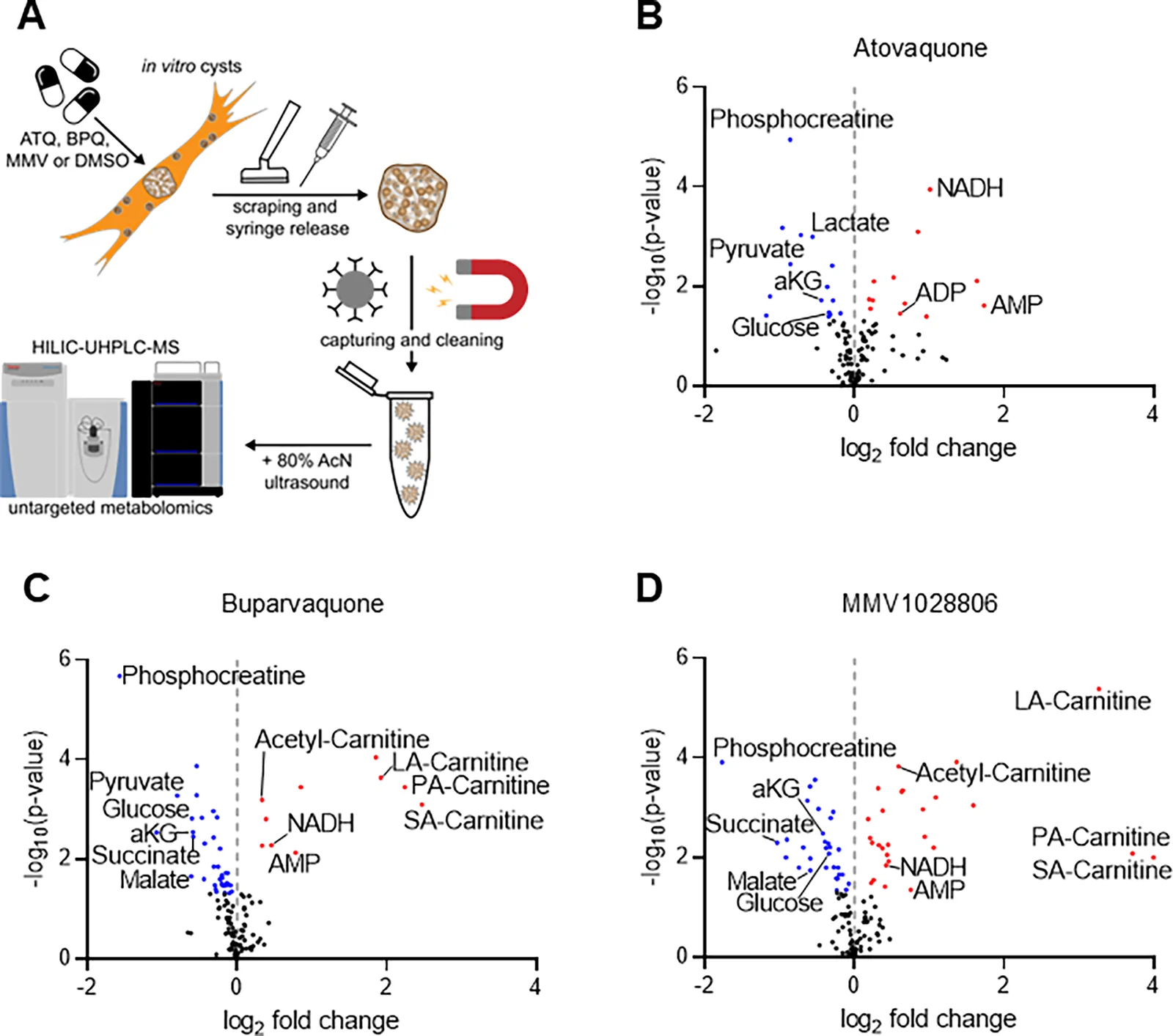
Untargeted metabolomic analysis of bradyzoites treated with *bc*_1_-complex inhibitors shows an energy imbalance. (**A**) Four-week-old in vitro cysts were treated for 3 hr. The cell monolayer was scraped off and the cysts were syringe-released. With *Dolichos biflorus* agglutinin (DBA) coated magnetic beads, the isolated cysts were captured and washed with PBS. The metabolites were extracted with 80% acetonitrile (AcN) in water and sonication, and analyzed via hydrophilic-interaction ultra-high pressure liquid chromatography coupled mass spectrometry (HILIC-UHPLC-MS). The results are shown in three separate volcano plots: Metabolic phenotypes of bradyzoites treated with atovaquone (**B**), buparvaquone (**C**), and MMV1028806 (**D**). (n=3, significance analyzed with a two-sided Mann-Whitney U test). Abbreviations: ATQ, atovaquone; BPQ, buparvaquone; DMSO, dimethyl sulfoxide; LA-carnitine, linoleoylcarnitine; MMV, MMV1028806; NADH, reduced nicotinamide adenine dinucleotide; PA-carnitine, palmitoylcarnitine; SA-carnitine, stearoylcarnitine.

Next, we sought to confirm this mode of action directly on bradyzoites and record the metabolic impact of ATQ, BPQ, or MMV1028806. To that end, we exposed intracellular 4-week-old cysts to 5 µM, 500 nM, or 5 µM of the respective compounds for 3 hr and used 0.2% DMSO as a mock treatment. These concentrations represent their LC_50_ against bradyzoites and were chosen to minimize off-target effects. The cysts were isolated for LCMS analysis using magnetic beads (Christiansen et al., 2022; Figure 6A, Supplementary file 5). Common in all three distinct metabolic phenotypes (Figure 6—figure supplement 1) is the accumulation of AMP and NADH, while varying TCA cycle intermediates, such as malate, succinate, and α-ketoglutarate, and mitochondria-independent energy sources, such as glucose and phosphocreatine, were depleted (Figure 6B–D). These metabolic changes suggest an energy starvation scenario marked by rising AMP levels and reduced recycling of NADH via the *bc*_1_-complex. Interestingly, in BPQ- and MMV1028806-treated cysts, we also observed prominent accumulation of several acyl-carnitine species which are mobile intermediates of β-oxidation (Figure 6B–D). Since this pathway is thought to be absent in *T. gondii,* we attribute these changes to potential inhibitory effects on host mitochondria. Notably, we did not observe changes in the pyrimidine synthesis pathway, which may reflect low nucleic acid demand from largely dormant bradyzoites. These data demonstrate that bradyzoites, which were considered metabolically inactive, exhibit metabolic responses to inhibitory compounds. Although we characterized AMP accumulation as part of a general stress response in tachyzoites, the metabolic profiles of inhibited bradyzoites are consistent with inhibition of their mitochondrial *bc*_1_-complex and suggest its role in ATP generation.

**Figure 6—figure supplement 1.**
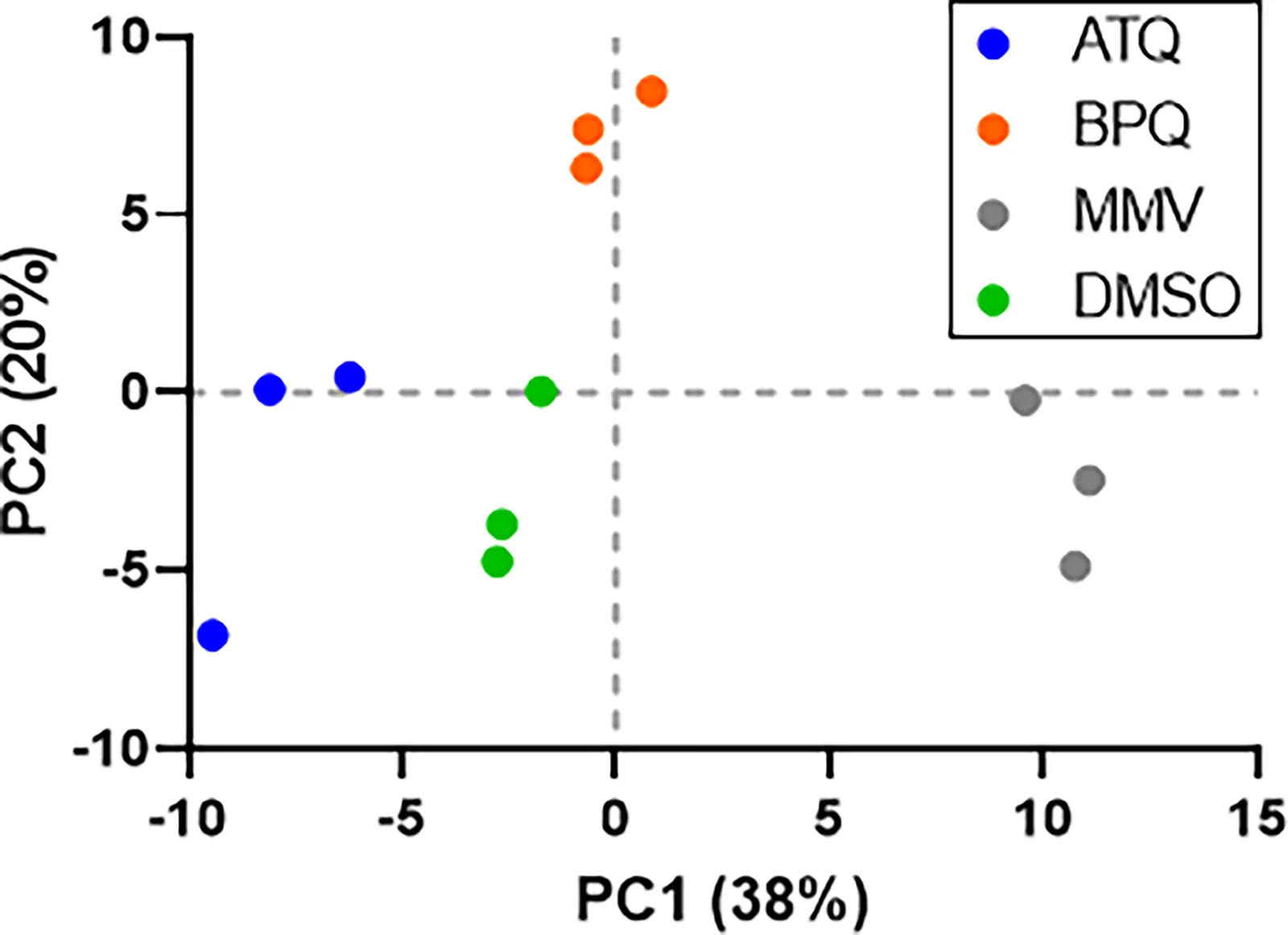
Principal component analysis of bradyzoites treated with atovaquone (ATQ), buparvaquone (BPQ), and MMV1028806. Four-week-old intracellular cysts were exposed for 3 hr, purified from their host cells using magnetic beads, and analyzed by LCMS.

### The differential efficacy of ATQ and HDQ against bradyzoites correlates with the inhibition of ATP levels in bradyzoites

**Figure 7.**
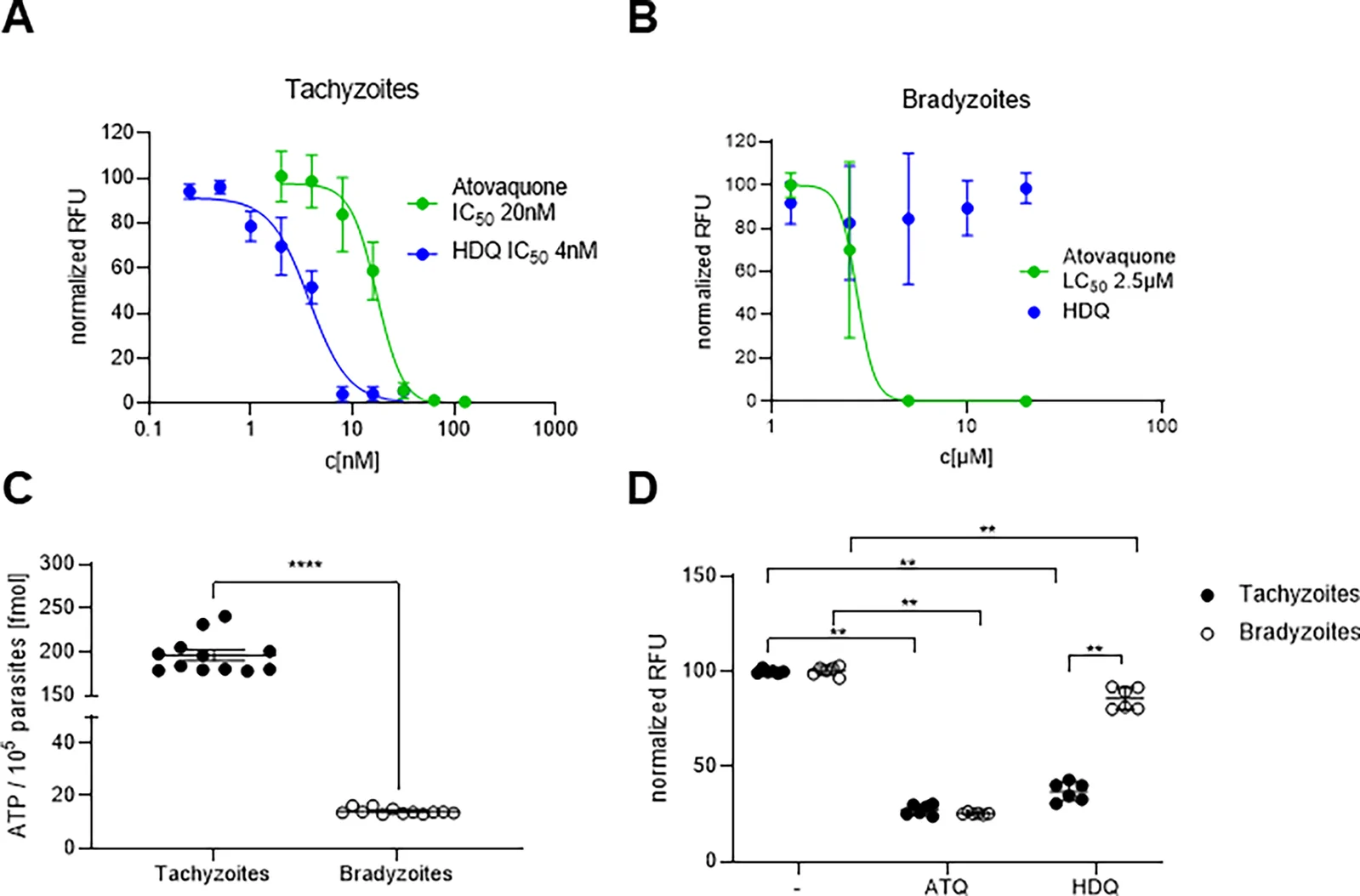
Differential efficacy and ATP depletion by atovaquone and 1-hydroxy-2-dodecyl-4(1H)quinolone (HDQ) in tachyzoites and bradyzoites. Atovaquone and HDQ were tested against *T. gondii* Pru parasites. (**A**) Showing the normalized half-maximal inhibitory concentration (IC_50_) against tachyzoites in fibroblasts at day 4, n=8. (**B**) 4-week-old bradyzoites were treated for a week with atovaquone or HDQ. Cultures were observed for tachyzoite regrowth for 3 weeks. The half-maximal lethal concentration (LC_50_) was determined at the time point which ideally reflected the dose-response curve, in this case day 14, n=6. 10^5^ tachyzoites and bradyzoites were lysed and the ATP concentration was determined with an enzymatic luciferase assay. (**C**) Showing the calculated concentration of ATP within an average parasite. Lines reflect the median, error bars represent SEM, significance test Mann-Whitney, n=12. (**D**) Parasites were treated with 1 μM atovaquone or HDQ. RFUs were normalized to the untreated control of either the tachyzoite or bradyzoite dataset. Shown are means, error bars represent SD, significance test is pairwise Mann-Whitney, n=6.

We sought to test the importance of the mETC chain in ATP generation in bradyzoites and exploited the differential targets of the coenzyme Q analogs ATQ and HDQ. However, while ATQ inhibits the *bc*_1_-complex, HDQ inhibits DHODH (Hegewald et al., 2013) and the alternative NADH dehydrogenase (NDH2) (Saleh et al., 2007). Both compounds block tachyzoite growth at low nanomolar concentrations in vitro with IC_50_s at 20 and 4 nM, respectively (Figure 7A). However, in contrast to ATQ, HDQ does not affect the viability of mature *T. gondii* bradyzoites despite diminishing their mitochondrial membrane potential (Christiansen et al., 2022). Up to 20 µM HDQ did not arrest re-differentiation into proliferating tachyzoites, while bradyzoites that were treated with 5 µM ATQ (LC_50_ 2.5 µM) did not generate proliferating tachyzoites (Figure 7B). To investigate how these modes of actions affect the mETC in bradyzoites, we tested their influence on mitochondrial ATP production by luciferase-based ATP assay. This assay shows a linear response to parasite cell equivalents between 10^3^ and 10^6^ parasites per 100 μL, and in order to minimize variance we decided to work with 10^5^ parasites (Figure 7—figure supplement 1). To maximize mitochondrial ATP production, we starved 3-week-old bradyzoites for 1 additional week of glucose. As a comparison, we utilized tachyzoites that were cultured for 2 days in glucose-deplete conditions. Untreated tachyzoite samples contained on average 196±21 fmol ATP per 10^5^ parasites, whereas untreated bradyzoites only contained 14.1±1.1 fmol (Figure 7C). Three-hour-long treatment with 5 µM ATQ decreased ATP levels in both stages by 75% (Figure 7D). In contrast, HDQ reduced ATP levels by 60% in tachyzoites but only marginally affected bradyzoites (Figure 7D). These data indicate that bradyzoites can maintain very low ATP levels and suggest the presence of a diminished but obligate production of ATP in the mitochondria that functions independently of exogenous glucose and HDQ-target enzymes.

**Figure 7—figure supplement 1.**
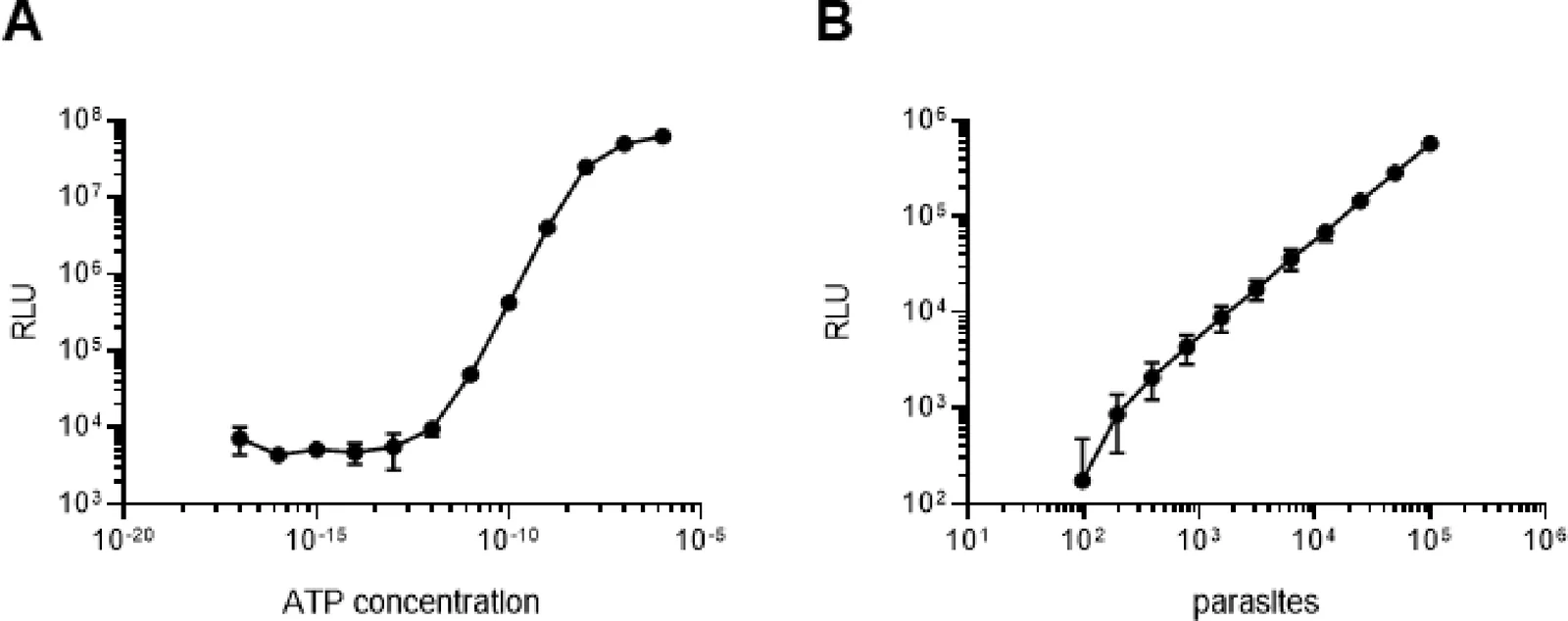
Luminescence-based ATP assay. (**A**) The linear response of the BacTiter-Glo assay has a certain range, depending on the ATP concentration of the sample. The assay is reliable between 100 fM and 1 nM ATP. (**B**) Increasing numbers of freshly isolated parasites were analyzed to determine a minimal parasite density necessary for steady readouts. 10^5^ parasites per sample were both manageable and accurate.

## Discussion

New treatment options for chronic *T. gondii* infections are needed, as chronic infections are currently incurable and available treatments for acute toxoplasmosis are associated with severe side effects, in particular in infants after congenital transmission (Konstantinovic et al., 2019). To simultaneously identify drug targets in bradyzoites and along with their inhibitory compounds, we utilized a recently developed in vitro culture system of pan-drug tolerant bradyzoites (Christiansen et al., 2022) to screen 400 compounds of the MMV Pathogen Box. 16 compounds exhibited confirmed cidal activity against bradyzoites while also inhibiting growth of tachyzoites. Metabolic profiling and stable isotope tracing in treated tachyzoites suggested the inhibition of the mitochondrial *bc*_1_*-*complex by MMV1028806 and the reference compound BPQ.

Interestingly, of the 15 compounds in the toxoplasmosis disease set, previously earmarked by MMV, only 10 were found to be active against tachyzoites and none against bradyzoites. These differences may be attributed to strain-specific susceptibilities which are known for anti-folates and ATQ (Meneceur et al., 2008) and differences in the design of growth assays. MMV assessed activities via plaque assays that prevent media change and rely on visual inspection of growth of a low number of parasites and might overestimate inhibitory effects.

We found that bradyzocidal compounds exhibit higher predicted logP coefficients, suggesting that the parasite entry might occur via diffusion across the cyst wall and less via solute transporter proteins. A known example of this principle is the anti-malarial drug fosmidomycin, where high polarity and low import rates caused minimal uptake by and efficacy against *T. gondii* tachyzoites (Nair et al., 2012; Baumeister et al., 2011). A similar case might also be made for the antifolates, pyrimethamine, sulfadiazine, trimethoprim, and sulfamethoxazole, which exhibit low consensus logP values of 2.09, 0.4, 1.16, and 0.92 and which also fail to eradicate tissue cysts (Alday and Doggett, 2017; Enshaeieh et al., 2021). Interestingly, MMV675968 is a dually active compound with a logP value of 2.46, which has been shown to be a potent inhibitor of the dihydrofolate reductase (DHFR) in a range of pathogens (Songsungthong et al., 2019; Songsungthong et al., 2021; Kim et al., 2021; Borba-Santos et al., 2020; Nugraha et al., 2019; Rollin-Pinheiro et al., 2021; Vila and Lopez-Ribot, 2017; Cantillon et al., 2022). Its target in *T. gondii*, however, remains to be identified.

Another important diffusion barrier that needs to be overcome to eradicate *T. gondii* tissue cysts is the bbb. Pyrimethamine and trimethoprim penetrate the bbb (Geils et al., 1971; Ineichen et al., 2020); however, they do not clear brain-resident cysts (Montazeri et al., 2018; Enshaeieh et al., 2021; Christiansen et al., 2022), highlighting the cyst wall as a potentially decisive obstacle. Consistent with this hypothesis is the tolerance of mature in vitro tissue cysts against high doses of sulfadiazine and pyrimethamine (Christiansen et al., 2022).

To characterize the modes of action of four additional inhibitors, we used metabolomics. In general, metabolic phenotypes reflect direct consequences of target inhibition as well as indirect effects resulting from attenuated growth and stress responses. We chose the bumped-kinase inhibitor MMV688854 (BKI-1294) that targets the calcium-dependent protein kinase 1 (CDPK1) as a control compound to identify metabolites implicated in a general stress response and adaptation to slow growth (Winzer et al., 2015). CDPK1 is needed for invasion (Johnson et al., 2012) and also affects formation of daughter cells. An accumulation of AMP and oxidized glutathione are the most conserved responses across the six treatments and may indicate depleted energy levels and oxidative stress (Caro et al., 2012). Also, pseudo uridine is elevated in many treatments. Enhanced pseudo uridylation of RNA is considered to be implicated in stress responses (Cerneckis et al., 2022) and modulates RNA stability (Davis, 1995). It remains to be tested which of these conserved metabolic responses signify cellular decay and which ones take part in a productive metabolic response in *T. gondii*. Nonetheless, metabolic profiling data on antibiotics-exposed bacterial pathogens has been used to design combination therapies, either by interfering with productive metabolic stress responses (Zampieri et al., 2017) or by simultaneously inhibiting otherwise non-essential pathways (Campos and Zampieri, 2019). We found that the systematic identification of target structures and drug modes of action from profiling data alone remains challenging due to inherent nonlinear relationships within the cellular metabolic network, the underlying regulatory mechanisms of enzyme functions. A systematic mapping of genetic disruptions to metabolic phenotypes, as demonstrated in *Escherichia coli*, may alleviate these restrictions (Anglada-Girotto et al., 2022).

Apart from the general metabolic stress response to the treatment with MMV688854, three compounds caused apparent inhibitor-specific signatures. MMV019189, which is known to inhibit *P. falciparum* (Duffy et al., 2017), but also *C. parvum* (Hennessey et al., 2018) and *Leishmania mexicana* (Berry et al., 2018), led to the accumulation of c-GMP-AMP in the parasite. This dinucleotide is produced by the c-GMP-AMP synthase (cGAS) in mammalian cells in response to cytosolic DNA and triggers a type-I interferon response (Sun et al., 2013). While cGAS is considered absent in *T. gondii*, host c-GMP-AMP induces STING-signaling as a response to *T. gondii* infection (Wang et al., 2019). Hence, MMV019189 may implicate the host in its antiparasitic activity.

MMV228911-treated tachyzoites showed the depletion of the DXR product MEP, a metabolite that occurs in the non-mevalonate pathway in *T. gondii*’s apicoplast. Other metabolites upstream of DXR are either not detected or depleted, such as glyceraldehyde 3-phosphate, but also partake in other pathways. Interestingly, MMV228911 also caused a broader dysregulation of central carbon metabolism marked by a decrease in several phosphorylated metabolites, including nucleotide triphosphates and PEP, as well as citric acid and acetyl-CoA. This is consistent with metabolic signaling beyond the apicoplast, which also has been implicated in *P. falciparum*, where a cytosolic sugar phosphatase is responsible for resistance to the DXR inhibitor fosmidomycin (Guggisberg et al., 2014).

We identified MMV1028806 as a candidate *bc*_1_-complex inhibitor by comparing its metabolic impact with that of BPQ and ATQ. All three compounds cause a sharp increase in pyrimidine synthesis substrates, depletion of pyrimidines, and dysregulation of TCA cycle intermediates. This is consistent with previous mass spectrometry-based studies that characterized the metabolic fingerprint of ATQ on *P. falciparum* intraerythrocytic blood stages (Creek et al., 2016; Allman et al., 2016; Antonova-Koch et al., 2018). In addition to abundance data, the incorporation of ^13^C and ^15^N stable isotopes from glucose and glutamine, respectively, into TCA cycle and pyrimidine biosynthesis intermediates suggests the *bc*_1_-complex as a target. Both the isotopologue distributions and metabolite abundances strongly suggest attenuation of pyrimidine production and TCA cycle fluxes by all three inhibitors (Hortua Triana et al., 2016). TCA cycle intermediates show characteristic isotopologue distributions that are indicative of ^13^C-label incorporation via acetyl-CoA (MacRae et al., 2012). A relatively increased M+3 isotopologue species in dicarboxylic acids in ATQ- and BPQ-treated parasites may indicate enhanced anaplerotic synthesis from glycolytic intermediates. These distinctions from MMV1028806 metabotypes may reflect a combination of off-target effects and differential timing of the metabolic inhibition.

Interestingly, six compounds from the MMV Pathogen Box have recently been shown to inhibit O_2_ consumption in tachyzoites through *bc*_1_-complex inhibition (Hayward et al., 2023). We co-identified BPQ as a *bc*_1_-complex inhibitor, but we did not test the remaining five compounds for their targets. However, in our screen at 10 µM, we found MMV024397 and MMV688853 to be dually active, while trifloxystrobin (MMV688754) and auranofin (MMV688978) lacked inhibitory activity in bradyzoites, and azoxystrobin (MMV021057) was inactive. In addition, the previously described *bc*_1_-complex inhibitor ELQ400 (MMV671636) (McConnell et al., 2018) was dually active. Overall, these data are consistent with the fact that the *bc*_1_-complex is indeed needed for the survival of bradyzoites, while not every tachyzoidal *bc*_1_-inhibitor also inhibits bradyzoites.

In contrast to tachyzoites, the metabolic phenotype in bradyzoites is not dominated by DHODH (Figure 6B, C, and D), as we observed neither the accumulation of carbamoyl-aspartate, dihydroorotate, and aspartate, nor the depletion of pyrimidines. Instead, AMP and NADH accumulate, which in eukaryotes signifies a low cellular energy status (Lin and Hardie, 2018; Fu et al., 2019) that typically induces AMP-activated protein kinase and a glycolytic response. AMPK is a conserved enzyme with a pivotal role in cellular homeostasis: it responds to elevated AMP levels by repressing anabolic activity and by stimulating glucose uptake and glycolysis, β-oxidation, and ketogenesis (Winder and Hardie, 1999; Lin and Hardie, 2018). *T. gondii* tachyzoites express AMPK, which serves an important role in metabolic reprogramming during the lytic cycle of tachyzoites (Li et al., 2023). Transcriptomic and proteomic data indicate its presence in bradyzoites (Garfoot et al., 2019), but its function remains unknown. We observed decreased levels of free glucose during all three treatments that are consistent with stimulated glycolysis. We also observe depleted phosphocreatine levels in bradyzoites (Figure 6) during treatments. In muscle cells, phosphocreatine acts as an ATP buffer system and replenishes ATP pools from ADP via creatine kinase (Kuby et al., 1954; Perry, 1954). It remains to be seen whether bradyzoites possess this mechanism. We also detect elevated levels of acyl-carnitines in BPQ- and MMV1028806-treated bradyzoites. These molecules act as shuttles for the mitochondrial import of fatty acids for β-oxidation. However, this pathway has not been shown to be active and is deemed absent in *T. gondii* (Seeber et al., 2008; Shunmugam et al., 2022). The presence of acyl-carnitines in bradyzoites might reflect import from the host. It is conceivable that their elevation in response to BPQ and MMV1028806 indicates compromised functionality of the host *bc*_1_-complex and subsequently accumulating β-oxidation substrates. Indeed, BPQ has a very broad activity across Apicomplexa (Hudson et al., 1985) and kinetoplastids (Croft et al., 1992).

We investigated the functionality of the mETC of tachyzoites and bradyzoites in response to ATQ, BPQ, and MMV1028806 by MitoTracker staining and thin section electron microscopy. All three treatments decrease overall MitoTracker fluorescence, consistent with a specific decrease in mitochondrial electron potential (Figure 5—figure supplement 1). In contrast, pyrimethamine, clindamycin, and DON did not reduce the MitoTracker signal. Pyrimethamine-treated samples displayed an increased MitoTracker-to-DAPI ratio, which can be attributed to diminished DAPI intensity, likely resulting from inhibition of folate-dependent DNA synthesis via DHFR blockade. Clin-treated samples showed no change in mitochondrial morphology or ratio, in line with its delayed-death phenotype. Similarly, DON, which targets glutamine metabolism, did not alter mitochondrial signal or ratio at 24 hr, though morphological changes were observed. Together, these findings confirm that disruption of mitochondrial membrane potential is specific to compounds targeting the electron transport chain, while inhibitors acting on nuclear or metabolic pathways do not induce rapid mitochondrial dysfunction under the conditions tested. ATQ and MMV1028806, but not BPQ, also shrink areas of mitochondrial cross-sections, indicating a potential contribution to the observed change in overall fluorescence. These differences might reflect distinct mitochondrial morphologies. In agreement with previous observations (Place et al., 2023), we found that bradyzoite mitochondria differ from those in tachyzoites, predominantly occurring as ‘blobs’ rather than tubes or loops, and our observations align with these findings (Figure 5—video 1).

The oxygen consumption assays highlight the functional sensitivity of the *T. gondii* RH∆ku80 mETC to pharmacological perturbation (Figure 5E). As expected, FCCP, an uncoupler of oxidative phosphorylation, markedly increased oxygen consumption, confirming the parasite’s ability to upregulate electron flow when the proton gradient is dissipated. Antimycin A nearly abolished respiration, demonstrating the essential role of complex III in maintaining mitochondrial activity. Likewise, ATQ and BPQ, both *bc*_1_-complex inhibitors, strongly suppressed oxygen consumption. Notably, MMV1028806 produced a comparable inhibitory effect, suggesting that it also targets complex III or a functionally linked site within the mitochondrial electron transport chain. This observation differs from the findings of Hayward et al., 2023, who did not report MMV1028806 as an mETC inhibitor in their Seahorse-based screen of the MMV Pathogen Box. A key difference is that our assays were performed at 10 µM, whereas Hayward et al., 2023, used 1 µM. These results support a mitochondrial mode of action for MMV1028806 under our experimental conditions and underline the value of oxygen consumption assays for detecting dose-dependent effects on parasite respiration.

Our findings strongly suggest that bradyzoite survival is critically dependent on mitochondrial electron transport. We investigated this by comparing the effects of two inhibitors: HDQ, which kills tachyzoites but not bradyzoites, and ATQ, which kills both. Although HDQ enters bradyzoites and reduces their mitochondrial membrane potential, its inability to kill them implies its molecular targets are non-essential in this stage. To pinpoint the vital process, we measured ATP levels and found that only the bradyzocidal compound, ATQ, caused a collapse in bradyzoite ATP. HDQ did not, despite both drugs depleting ATP in tachyzoites. This directly links bradyzoite viability to mitochondrial ATP production. The different outcomes are explained by their distinct targets: ATQ inhibits the *bc*_1_-complex (McFadden et al., 2000), which is central to ATP synthesis, whereas HDQ blocks other pathways like pyrimidine synthesis and mitochondrial NADH oxidation through DHODH and NDH2. These results also support the notion that the canonical TCA cycle is not the primary source of electrons for bradyzoites (Christiansen et al., 2022). A variety of electron sources supply the electron transport chain (Maclean et al., 2021). The sources that are crucial for ATP production remain to be identified.

Bradyzoites may also utilize glycolysis to generate ATP. This has been suggested to be the case in early, 7-day-old bradyzoites where gliding motility, an ATP-dependent process, can be inhibited by withdrawal of exogenous glucose and by the glycolytic inhibitor 2-deoxyglucose. However, in these parasites, also oligomycin A inhibited motility, suggesting a simultaneously essential contribution of the mitochondrial ATP (Fu et al., 2021). In addition, hexokinase has been shown to be needed for optimal cyst production in chronically infected mice, suggesting an important role of this protein either through its enzymatic activity or other functions (Shukla et al., 2018). It remains to be investigated how mature bradyzoites rely on glycolysis to produce ATP and how this is integrated with mitochondrial ATP production.

Interestingly, despite its profound effect on ATP levels and viability of bradyzoites, ATQ fails to eradicate all *T. gondii* cysts from infected mice and does not reliably prevent toxoplasmosis relapse in AIDS patients and mice (Araujo et al., 1991; Kovacs, 1992; Ferguson et al., 1994; Katlama et al., 1996b). Underlying reasons for this discrepancy need to be established but may include insufficient bioavailability. Quinolones are known to be poorly soluble in aqueous environments. In addition, the predicted permeability of the bbb to either ATQ, BPQ, and HDQ is low. Indeed, improved bioavailability of ATQ in nanosuspensions improved efficacies (Shubar et al., 2011). Also, BPQ treatment of mice that were experimentally infected with closely related *N. caninum* parasites did target parasites in muscle but not in brain tissue (Müller et al., 2015). A similar conjecture arose when comparing endochin-like quinolones. Here, favorable pharmacokinetics of a variant with lower intrinsic potency likely conferred higher in vivo efficacy against cysts (Doggett et al., 2012).

Together, our data illustrate the functional importance of the *bc*_1_-complex for the viability of bradyzoites and encourage the pursuit of *bc*_1_-complex inhibitors as candidates for curative toxoplasmosis treatment. Both efforts to develop multi-site-directed inhibitors (Doggett et al., 2020; Alday et al., 2022) and re-purposing of respective drugs are promising avenues. However, our data also underline the importance of favorable pharmacokinetics to ensure passage through the bbb and the cyst wall.

## Materials and methods

**Key resources table:** 

Reagent type (species) or resource	Designation	Source or reference	Identifiers	Additional information
Strain, strain background (*Toxoplasma gondii*)	PruTom Prugniaud ∆hxgprt tdTomato	John et al., 2009 PMID:19578440	RRID:NCBITaxon_748168	
Strain, strain background (*Toxoplasma gondii*)	ME49	Suzuki et al., 1989 PMID:2926171	RRID:NCBITaxon_748168	
Strain, strain background (*Toxoplasma gondii*)	RH-Rep1/2-S9(33–159)-GFP (RH-S9)	DeRocher et al., 2000; Thomsen-Zieger et al., 2003; PMID:11058084; PMID:12860390	RRID:NCBITaxon_748168	
Biological sample (*Homo sapiens*)	BJ-5ta human foreskin fibroblasts (HFF)	ATCC	Cat#: CRL-4001 RRID:CVCL_6573	Synonymous with BJ1-hTERT
Biological sample (*Homo sapiens*)	Human myoblast cells (KD3)	Shiomi et al., 2011; PMID:21490680		Immortalized primary cells. Availability statement here: PMID:38213326
Chemical compound, drug	MMV Pathogen Box	Medicines for Malaria Venture		Provided by MMV free of charge
Software	ImageJ v1.54	Schindelin et al., 2012; PMID:22743772	RRID:SCR_003070	Version 2.16.0
Software	GraphPad Prism	RRID:SCR_002798		Version 8.4.1
Software	Compound Discoverer	Thermo Fisher	RRID:SCR_028693	Version 3.1
Other	DAPI stain	Sigma-Aldrich	Cat#: D9542	More information in the Fluorescence stain of mitochondrial activity section
Other	Streptavidin-Cy2	Jackson Antibodies	Not available	More information in the Fluorescence stain of mitochondrial activity section
Other	*Dolichos biflorus* agglutinin	Sigma-Aldrich	Cat#: L6533	More information in the Fluorescence stain of mitochondrial activity section
Other	MitoXpress Xtra Oxygen Consumption Assay	Agilent Technologies	Cat#: MX-200-4	Following the manufacturer’s high-sensitivity (HS) method with modifications. More information in the Fluorescence stain of mitochondrial activity section

### Tachyzoite culture

Prugniaud ∆hxgprt tdTomato (John et al., 2009) (PruTom), RH-Rep1/2-S9(33–159)-GFP (DeRocher et al., 2000; Thomsen-Zieger et al., 2003) (RH-S9) and ME49 (Suzuki et al., 1989) parasites were maintained in HFF cells (BJ-5ta HFF ATCC, CRL-4001) with Dulbecco’s modified Eagle’s medium (DMEM) containing 25 mM glucose, 4 mM L-glutamine, 1 mM sodium pyruvate, 100 U/mL penicillin, 100 µg/mL streptomycin, and 1% heat-inactivated fetal bovine serum (FBS, Gibco) at 37°C with 10% CO_2_ (Christiansen et al., 2022). All host and parasite cell lines and primary cells tested negative for mycoplasma contamination at the time of import into the laboratory and twice a year from there on. Identity of *T. gondii* lines was confirmed phenotypically via their fluorescence and drug resistance. The identity of all strains was confirmed by Illumina-based whole-genome sequencing once. Identity of BJ-5ta cells was verified microscopically.

### In vitro *T. gondii* tissue cyst culture

The *T. gondii* in vitro cyst culture was performed as in Christiansen et al., 2022; Maus et al., 2024. In short, KD3 myoblasts (Shiomi et al., 2011) were grown to 70% confluency, followed by the induction of myotube differentiation by changing the medium to DMEM as described above but with 2% horse serum instead of FBS and additionally supplemented with 10 µg/mL human insulin, 5 µg/mL human holo-transferrin, and 1.7 ng/mL Na_2_SeO_3_. After 5 days, the myotubes were infected with PruTom (MOI 0.1) in bicarbonate-free Roswell Park Memorial Institute (RPMI) medium containing 5.5 mM glucose, 50 mM HEPES, 4 mM L-glutamine, 100 U/mL penicillin, 100 µg/mL streptomycin, 2% horse serum, 10 µg/mL human insulin, 5 µg/mL human holo-transferrin, and 1.7 ng/mL Na_2_SeO_3_ at pH 7.2. Cultures were incubated at 37°C at ambient CO_2_ for 4 weeks and the medium was changed every 2–3 days. A comprehensive scheme of the procedure is shown in Figure 1—figure supplement 1. The identity of KD3 cells was confirmed via their drug resistances (G418 at 400 µg/mL and puromycin at 0.5 µg/mL) and morphologies of myoblasts and myotubes.

### MMV Pathogen Box screening procedure

All Pathogen Box compounds were stored at –20°C (Veale, 2019). DMSO-solved compounds were diluted in DMEM or RPMI media and stored at 4°C until use. Host cells were grown in transparent, flat-bottom 96-well plates and infected as described above to cultivate either tachyzoites or in vitro tissue cysts. One column per plate was left uninfected to estimate fluorescence background. Four hours after infection with tachyzoites or after 4 weeks of bradyzoite maturation, the culture medium was replaced by medium containing either 0.1% DMSO (negative control) or 10 µM of the respective Pathogen Box compound. During the 7-day treatment period, the medium was renewed every 2–3 days. It was then changed to tachyzoite growth medium to induce re-differentiation of viable bradyzoites to tachyzoites, and then the culture was monitored over 28 days. Prior to each medium exchange, the cultures were monitored for compound crystallization. The fluorescence was measured in a fluorescence plate reader (Tecan Infinite M200 PRO) (excitation Λ=554 nm, emission Λ=589 nm). Fluorescence values passing a threshold of 2000 after background subtraction indicated proliferating parasites and hence an ineffective treatment (Figure 1).

### Resazurin viability assay and cytotoxicity

After the growth assays were completed, the host cell monolayers of both HFF and KD3 myotubes were washed with PBS and incubated with 500 nM resazurin in Milli-Q water at 37°C for 4 hr. Fluorescence was measured (excitation Λ=540 nm, emission Λ=580 nm) in a fluorescence plate reader and normalized to the average fluorescence values of the DMSO-treated controls, which were set at 100%. During bradyzoite assays, KD3 host cells were visually inspected after drug exposure for 1 week. After completion of the regrowth phase for 4 weeks, the colorimetric resazurin was performed. These values were reported as lowest cytotoxicity values (Figure 2—figure supplement 1). During secondary tests of putatively dually active compounds, resazurin assays were performed in a concentration-dependent manner (Supplementary file 1, Figure 2—figure supplement 1).

### The dose response of dually active compounds

For the measurement of LC50 and MLCs, tachyzoite and bradyzoite cultures were treated with the MMV compounds which were pipetted in a serial dilution across the 96-well plate and treated for 7 days. Afterward, the compound containing medium was removed and possible regrowth was monitored for another 7 days (tachyzoite assay) or 21–28 days (bradyzoite assay) in a fluorescence plate reader (Tecan Infinite M200 PRO) (excitation Λ=554 nm, emission Λ=589 nm). The relative fluorescence units were background-subtracted. Depending on the development of the DMSO control, a late time point when DMSO-treated cultures still continued exponential growth was chosen to calculate the half-maximal inhibitory concentration (IC_50_) for tachyzoites. This time point was between days 4 and 7. Bradyzoite cultures were of lower MOI, and we calculated LC_50_ values between experiments from 7 to 28 days post treatment when exponential growth occurred in mock-treated cultures (Figure 2). The concentration data was log-transformed and a nonlinear regression (curve fit with variable slope) was performed to determine the dose response (Prism 9, GraphPad).

### In silico predictions

MMV provided the SMILES IDs for every compound, which were compiled with LightBBB (Shaker et al., 2021) to predict the blood-brain permeability. SwissADME (Daina et al., 2017) was used to predict the gastrointestinal absorption capability. The logP values (Figure 2—figure supplement 1) were provided by MMV, and remaining logP values were calculated on SwissADME (Daina et al., 2017) as consensus values.

### Fluorescence stain of mitochondrial activity

This assay was performed on tachyzoites and bradyzoites. For the tachyzoite experiment, HFF cells were infected with RH-S9 parasites at an MOI of 0.1 and treated for 24 hr with 1 µM ATQ, 25 nM BPQ, or 5 µM MMV1028806, respectively, corresponding to fivefold of the respective IC_50_ values, or 0.1% DMSO as a solvent control. For the bradyzoites experiment, 4-week-old ME49 in vitro cysts were treated for 24 hr with 5 µM ATQ, 500 nM BPQ, or 5 µM MMV1028806, respectively, corresponding to the lowest lethal concentration, or 0.1% DMSO as a solvent control.

The assay was repeated with additional control treatments (Figure 5—figure supplement 1). In this experiment, HFF cells were infected with RH-S9 tachyzoites and, 4 hr post-infection, incubated for 24 hr with 0.1% DMSO (solvent control), 1 µM ATQ, 25 nM BPQ, 5 µM MMV1028806 (MMV), 1 µM pyrimethamine (Pyri), 10 nM clindamycin, or 25 µM DON. Concentrations reflected the fivefold IC_50_ values, except for DON, which does not inhibit growth.

Subsequent staining and imaging procedures were identical across all experiments. Cultures were then stained with 500 nM MitoTracker Deep Red FM (MT; Thermo Fisher) in DMEM for 40 min at 37°C, followed by a 20 min de-staining with DMEM and final fixation with 1% paraformaldehyde for 20 min at room temperature (RT). The cyst wall was decorated with *Dolichos biflorus* agglutinin (DBA) (Vector Laboratories, Cat# B-1035) and stained with Streptavidin-Cy2 (Jackson Antibodies, Cat# 016-220-084). Mowiol containing DAPI (1:3000) was used to mount the cells. To avoid a biased image acquisition, all parasite vacuoles or cysts identified in a specimen along a horizontal axis were imaged. Tachyzoite images were taken with a Zeiss Axio Imager Z1 Microscope equipped with the ApoTome 2 at identical exposure times (DAPI 200 ms, MT 500 ms, GFP 200 ms, Brightfield 10 ms) using a Zeiss Plan APOCHROMAT 63×/1.4 oil objective lens. Bradyzoite images were acquired on a Leica Stellaris 8 laser scanning confocal microscope using a Leica HC PL APO CS2 63×/1.4 oil objective lens. The pinhole diameter was set to 1 AU. All detectors were operating in photon counting mode. The acquisition parameters were set as follows: DAPI: diode laser at 405 nm_exc_ with 2.2%, 430–550 nm_em_ with a HyD S-detector. Cy2: WLL at 468 nm_exc_ with 8.5%, 473–591 nm_em_ with a HyD S-detector. Mitotracker: WLL at 641 nm_exc_ with 2.7%, 650–784 nm_em_ with a HyD X-detector (4× line average). The zoom was set to 7×, and images were acquired in frame-sequential mode with a unidirectional scan speed of 400 Hz; the pixel dwell time for all channels was 3.18 µs. For z-stacks, z-step size was set to 0.299 µm.

Using ImageJ (Schindelin et al., 2012), the intensity threshold of individual pixels of MT (190–255 for tachyzoites and 125–65,535 for bradyzoites) and DAPI (140–255 for tachyzoites and 363–65535) channels was cut off, and the signal intensity was calculated per parasitophorous vacuole or cyst.

### Oxygen consumption assay

To assess mitochondrial oxygen consumption in *T. gondii* RH∆ku80 tachyzoites, we employed the MitoXpress Xtra Oxygen Consumption Assay (Agilent Technologies, Cat# MX-200-4) following the manufacturer’s high-sensitivity (HS) method with modifications. Parasites were freshly harvested upon egress and resuspended at a density of 1×10^7^ tachyzoites per reaction in phenol red-free D1 medium lacking glucose but supplemented with glutamine to promote mETC activity via the GABA shunt (MacRae et al., 2012).

Parasites were incubated in medium with one of the following treatments immediately before measurement: 0.1% DMSO (vehicle control), 1  µM antimycin A (complex III inhibitor, positive control), 10 µM FCCP (uncoupler, commonly used to determine the maximum respiration capacity), 1 µM ATQ, 1 µM BPQ, or 1 µM MMV1028806. Untreated control wells contained 1×10^7^ tachyzoites in 0.1% DMSO. Background fluorescence was determined from wells containing MitoXpress Xtra reagent but no parasites.

Each 100 µL reaction was supplemented with 10 µL of reconstituted MitoXpress Xtra reagent. To prevent oxygen diffusion from ambient air, 100 µL of prewarmed HS mineral oil was carefully layered over each well. Plates were immediately transferred to a prewarmed Tecan Infinite 200 Pro plate reader, maintained at 37°C. Fluorescence was recorded every 2 min for 2 hr using the following settings: excitation at 380 nm, emission at 650 nm, gain 220, 25 flashes, 30 µs integration delay, and 100 µs integration time.

Data were analyzed by determining the slope of the fluorescence lifetime signal (indicative of oxygen consumption) after an equilibration period of 20 min. The linear portion of the curve (typically over a 60 min window) was used for slope determination. Background signal from cell-free wells was subtracted from all conditions to obtain net oxygen consumption rates. Data was normalized to the untreated control.

### Thin section electron microscopy

The *T. gondii* in vitro cyst culture was performed as described above. After 4 weeks, cells were treated with 5 µM ATQ, 500 nM BPQ, 5 µM MMV1028806, corresponding to the lowest lethal concentration, or 0.1% DMSO as a solvent control, for 24 hr. Cells were fixed by covering the monolayer with a fixation solution of 1% paraformaldehyde and 2.5% glutaraldehyde (Electron Microscopy Services) in 0.05 M HEPES buffer (pH 7.4). After incubation at RT for 3 hr, samples were sealed with parafilm and stored in the fridge until further processing. Fixed cells were scraped, sedimented by centrifugation (3000*×g*, 10 min) using a swing-out rotor, and washed twice with 0.05 M HEPES buffer. The cell sediment was embedded in low-melting-point agarose and stored in 2.5% glutaraldehyde in 0.05 M HEPES buffer. Small blocks of the embedded sediment were subjected to postfixation (osmium tetroxide, tannic acid), en bloc contrasting (uranyl acetate), dehydration, and embedding in epoxy resin according to a standard protocol (Laue, 2010).

Ultrathin sections were prepared with an ultramicrotome (UC7, Leica Microsystems, Germany) using a diamond knife (45°, Diatome, Switzerland). The sections were collected on bare copper grids (300 mesh, hexagonal mesh shape) or copper slot grids, contrasted with 2% uranyl acetate and 0.1% lead citrate, and coated with a thin (2–3 nm) layer of carbon. The ultrathin sections with a thickness of ~65 nm were examined with a transmission electron microscope (Tecnai Spirit, Thermo Fisher Scientific) operated at 120 kV. Images were recorded with a side-mounted CCD camera (Phurona, EMSIS, Germany) at 4095×2984 pixels.

### Metabolomic analysis of drug-treated intracellular tachyzoites and bradyzoites

HFF cells were infected with PruTom at MOI 1. Approximately 64 hr later, the cultures were treated with 1.5 µM MMV019189, 7 µM MMV1028806, 30 µM MMV228911, 1.2 µM MMV595321, 5 µM MMV688762, 2 µM MMV688854, respectively, and 0.1% DMSO as a control for 3 hr. These concentrations correspond to the fivefold respective IC_50_ values. Cultures were quenched in ice-cold PBS, and parasites were isolated from their host cells by passaging scraped monolayers through a 23G needle, filtered through a 3 µm polycarbonate filter, and, after microscopic counting, distributed at 1×10^8^ parasites per sample. Parasites were washed with ice-cold PBS via 5 min centrifugation at 21,500*×g* at 0°C. Metabolites were then extracted by sonication for 2 min in 50 µL 80% acetonitrile in water. The insoluble particles were pelleted for 5 min at 21,500*×g* at 0°C. 5 µL of each sample was collected to generate a pooled biological quality control, which was injected regularly within the otherwise randomized run list. 5 µL of each sample was injected onto either an ACQUITY UPLC BEH amide column (Waters) equipped with a VanGuard amide pre-column (Waters) running at 200 µL/min or onto a SeQuant ZIC-pHILIC (Merck) column with an OPTI-LYNX ZIC-pHILIC guard column cartridge (Optimize Technologies) running at 400 L/mL. Both columns were operated on a Vanquish Flex Instrument (Thermo Fisher). Analytes were chromatographically separated starting with 100% eluent A (90% acetonitrile in water with 10 mM ammonium carbonate) and 0% eluent B (10 mM ammonium carbonate in water), decreasing to 40% A and 60% B over 25 min. Metabolites were detected on a Q Exactive Plus (Thermo Fisher) equipped with an electrospray ionization source operated in rapid polarity-switching mode at an MS^1^ resolution of 70k and an MS^2^ resolution of 35k (Top3 MSX1). Peak extraction, retention time alignment, and peak annotation were computed with Compound Discoverer 3.1 (Thermo Fisher) using an in-house library of authentic standards and mzVault MS2 database, as detailed in Supplementary files 2–5. Blank subtraction, gap-filling, as well as statistical analysis were performed in Excel (Microsoft), and the data was plotted with GraphPad Prism 8.4.1. Principal component analysis was performed using ClustVis (Metsalu and Vilo, 2015).

### Isotopic labeling

Intracellular parasites were treated as mentioned above and incubated for 3 hr with DMEM containing either 25 mM U-^13^C-glucose or 4 mM ^15^N-amide-glutamine instead of the respective unlabeled carbon sources. Quenching, parasite isolation, metabolite extraction, and liquid chromatography were performed as described. To increase coverage of the isotopologues, metabolites were measured in positive and negative mode separately without MS^2^ scans but with an increased MS^1^ scan resolution of 140k. For peak identification purposes, the unlabeled DMSO-treated sample was analyzed with intermittent MS^2^ scans as described above.

### ATP luciferase assay

PruTom tachyzoites were cultivated in HFF cells without glucose, isolated 48 hr post-infection, and treated with 1 µM ATQ or HDQ for 3 hr. PruTom bradyzoites were matured for 3 weeks, glucose-starved for an additional week, and treated for 24 hr with 1 µM ATQ or HDQ before being released by needle passage 23G, pepsin digestion for 20 min at 37°C. The digestion reaction was stopped by neutralizing in 1.2% sodium bicarbonate (pH ≈ 8.3) with 0.0159 g/L phenol red as pH indicator dye (Christiansen et al., 2022). They were isolated by douncing on ice and filtered through a 3 µm polycarbonate filter. 10^5^ parasites were then aliquoted in 100 µL ice-cold PBS in a 96-well plate. ATP was quantified using BacTiter-Glo Microbial Cell Viability Assay (Promega) according to the manufacturer’s protocol using a standard curve. Data were plotted in GraphPad Prism 8.4.1.

### Metabolomic analysis drug responses on in vitro cysts

Intracellular cysts were isolated from their myotube host cells and extracted as described in Christiansen et al., 2022; Maus et al., 2024. In short, cysts were released by needle passage (23G) and captured with DBA-coated magnetic beads. PBS-washed cysts (with 2% BSA) were extracted in 80% acetonitrile and analyzed as described above. One dish yielded approximately 10^6^ cysts, which were distributed among three samples. Prior to a statistical analysis, the dataset was normalized by dividing the compound intensities through the summed intensities of the respective samples.

## Data Availability

Metabolomic data been deposited at MSV000100157 and are publicly available. The following dataset was generated: MausD BlumeM 2025Screening the MMV Pathogen Box reveals the mitochondrial bc1-complex as a drug target in mature *Toxoplasma gondii* bradyzoitesMASSIVE10.25345/C57941739PMC1358542442751871

## Additional files

Supplementary file 1. Screening data.

Supplementary file 2. Tachyzoite mass spectrometry profiling data and its curation.

Supplementary file 3. Tachyzoite ^13^C-labeling mass spectrometry data and its curation.

Supplementary file 4. Tachyzoite ^15^N-labeling mass spectrometry data and its curation.

Supplementary file 5. Bradyzoite mass spectrometry profiling data and its curation.

Supplementary file 6. Supplementary file containing abbreviations.

MDAR checklist
