# Supplementary material for: Screening the MMV Pathogen Box reveals the mitochondrial bc1-complex as a drug target in mature Toxoplasma gondii bradyzoites
Source: eLife. 2026 Sep 17;13:RP102511. doi: 10.7554/eLife.102511 (PMC13585424; doi:10.7554/eLife.102511)
Supplement: Supplementary file 2 [file elife-102511-supp6.docx]

AcCoA - Acetyl-CoA

AcCys – Acetylcysteine

AcPhe – Acetylphenylalanine

AMP – Adenosinemonophosphate

ATP – Adenosinetriphosphate

ATQ – Atovaquone

bbb - Blood brain barrier

BPQ - Buparvaquone

CarbAsp - Carbamoyl-aspartate

cGMP-AMP - cyclic Guanosinemonophosphate-adenosinemonophosphate

CMP – Cytosinemonophosphate

DHO - Dihydroorotate

DHODH - dihydroorotate dehydrogenase

DON - 6-diazo-5-oxonorleucine

FCCP - 4-(trifluoromethoxy)phenylhydrazone

GABA - gamma-Aminobutyratic acid

GluCys – Glutamylcysteine

GluGln – Glutamylglutamine

Gro1P - Glyceraldehyde 1-phosphate

Gro3P - Glyceraldehyde 3-phosphate

GSH - Glutathione oxidized, GSSG - Glutathione reduced

GTP – Guanosinetriphosphate

HDQ - 1-hydroxy-2-dodecyl-4(1H)quinolone

HP4 – Hexose phosphate No 4

HydKyn – Hydroxylkynurenine

IC_50_ - Half-maximal inhibitory concentration

MEP - 2-C-Methyl-D-erythritol 4-phosphate

mETC - Mitochondrial electron transport chain

NAcAla - N-Acetylalanine

NAcCys - N-Acetylcysteine

NAcMet - N-Acetylmethionine

NAcPhe - N-Acetylphenylalanine

OAcSer - O-Acetylserine

PalmCarn – Palmitoylcarnitine

PEP - Phosphoenolpyruvate, PseudoU - Pseudouridine

Trp – Tryptophan

UMP – Uridinemonophosphate

UTP – Uridinetriphosphate

Pyri – pyrimethamine
