# Supplementary material for: Screening the MMV Pathogen Box reveals the mitochondrial bc1-complex as a drug target in mature Toxoplasma gondii bradyzoites
Source: eLife. 2026 Sep 17;13:RP102511. doi: 10.7554/eLife.102511 (PMC13585424; doi:10.7554/eLife.102511)
Supplement: Peer review history

# Editor report: eLife Assessment

**DOI:** 10.7554/eLife.102511.4.sa0

**Authors:** Bavesh D Kana

**Affiliations:** University of the Witwatersrand South Africa

**Keywords:** Convincing, Valuable

This **valuable** study utilizes a newly developed approach to culture T gondii bradyzoites in myotubes, and then takes advantage of the antiparasitic compound collection known as the Pathogen Box, to find compounds that target both tachyzoite and bradyzoite forms of the parasite. A set of compounds yielding patterns consistent with targeting the mitochondrial bc1 complex was explored further, with **convincing** evidence for changes in ATP production in bradyzoites to support the conclusions about the importance of this complex. The paper will be interesting for parasitologists studying drug discovery of apicomplexan parasites.

# Referee report: Reviewer #1 (Public review)

**DOI:** 10.7554/eLife.102511.4.sa1

Summary:

The authors' goal was to advance the understanding of metabolic flux in the bradyzoite cyst form of the parasite *T. gondii*, since this is a major form of transmission of this ubiquitous parasite, but very little is understood about cyst metabolism and growth. This is an important advance in understanding and targeting bradyzoite growth.

Strengths:

The study used a newly developed technique for growing *T. gondii* cystic parasites in a human muscle-cell myotube format, which enables culturing and analysis of cysts. This enabled screening of a set of anti-parasitic compounds to identify those that inhibit growth in both vegetative (tachyzoite) forms and bradyzoites (cysts). Three of these compounds were used for comparative Metabolomic profiling to demonstrate differences in metabolism between the two cellular forms.

One of the compounds yielded a pattern consistent with targeting the mitochondrial bc1 complex, and suggest a role for this complex in metabolism in the bradyzoite form, an important advance in understanding this life stage.

Weaknesses:

Studies such as these provide important insights into the overall metabolic differences between different life stages, and they also underscore the challenge with interpreting individual patterns caused by metabolic inhibitors due to the systemic level of some of the targets. The authors have employed mock treatment and non-metabolic inhibitor controls to alleviate these challenges.

# Referee report: Reviewer #2 (Public review)

**DOI:** 10.7554/eLife.102511.4.sa2

Summary:

A particular challenge in treating infections caused by the parasite *Toxoplasma gondii* is to target (and ultimately clear) the tissue cysts that persist for the lifetime of an infected individual. The study by Maus and colleagues leverages the development of a powerful in vitro culture system for the cyst-forming bradyzoite stage of Toxoplasma parasites to screen a compound library for candidate inhibitors of parasite proliferation and survival. They identify numerous inhibitors capable of inhibiting both the disease-causing tachyzoite and the cyst-forming bradyzoite stages of the parasite. To characterize the potential targets of some of these inhibitors, they undertake metabolomic analyses. The metabolic signatures from these analyses lead them to identify one compound (MMV1028806) that interferes with aspects of parasite mitochondrial metabolism. In the revised version of the manuscript, the authors present convincing evidence that MMV1028806 targets the mitochondrial electron transport (ETC) chain of the parasite (although they don't identify the actual target in the ETC). The revised manuscript also nicely addresses my other criticisms of the original version. Overall, the study presents an exciting approach for identifying and characterizing much-needed inhibitors for targeting tissue cysts in these parasites.

Strengths:

The study presents convincing proof-of-principle evidence that the myotube-based in vitro culture system for *T. gondii* bradyzoites can be used to screen compound libraries, enabling the identification of compounds that target the proliferation and/or survival of this stage of the parasite. The study also utilizes metabolomic approaches to characterize metabolic 'signatures' that provide clues to the potential targets of candidate inhibitors. In addition to insights into candidate bradyzoite inhibitors, the study also provides new insights into the physiological role of the mitochondrial electron transport chain of bradyzoites, and raises a host of interesting questions around the functional roles of mitochondria in this stage of the parasite.

Weaknesses:

As noted in my previous review, the authors present convincing evidence that one of the compounds they have identified (MMV1028806) is targeting the mitochondrial electron transport chain (ETC). However, in the absence of an assay that directly measures bc1 activity (e.g. an enzymatic assay), they cannot be certain that it targets the bc1 complex in the ETC. I appreciate that the authors have toned down some of the conclusions around this. I do still think there are some places where the text is overstating the finding (noted below).

Line 30. "Stable isotope-resolved metabolic profiling on tachyzoites and bradyzoites identified the mitochondrial bc1-complex as a target of bradyzocidal compounds".

Line 546. "Metabolic profiling and stable isotope tracing in treated tachyzoites suggested the inhibition of the mitochondrial bc1-complex by MMV1028806 and the reference compound BPQ."

Line 622. "In addition to abundance data, the incorporation of ¹³C and ¹⁵N stable isotopes from glucose and glutamine, respectively, into TCA cycle and pyrimidine biosynthesis intermediates suggest the bc1-complex as a target."

# Referee report: Reviewer #3 (Public review)

**DOI:** 10.7554/eLife.102511.4.sa3

Summary:

The authors described an exciting 400-drug screening using a MMV pathogen box to select compounds that effectively affects the medically important Toxoplasma parasite bradyzoite stage. This work utilises a bradyzoites culture technique that was published recently by the same group. They focused on compounds that affected directly the mitochondria electron transport chain (mETC) bc1-complex and compared with other bc1 inhibitors described in the literature such as atovaquone and HDQs. They further provide metabolomics analysis of inhibited parasites which serves to provide support for the target and to characterise the outcome of the different inhibitors.

Strengths:

This work is important as, until now, there are no effective drugs that clear cysts during *T. gondii* infection. So, the discovery of new inhibitors that are effective against this parasite-stage in culture and thus have the potential to battle chronic infection is needed. The further metabolic characterization provides indirect target validation and highlight different metabolic outcome for different inhibitors. The latter forms the basis for new studied in the field to understand the mode of inhibition and mechanism of bc1-complex function in detail.

The authors focused in the function of one compound, MMV1028806, that is demonstrated to have a similar metabolic outcome to burvaquone. Furthermore, the authors evaluated the importance of ATP production in tachyzoite and bradyzoites stages and under atovaquone/HDQs drugs.

# Author comment: Author response

**DOI:** 10.7554/eLife.102511.4.sa4

**Authors:** Deborah Maus, Elyzana Putrianti, Tobias Hoffmann, Michael Laue, Frank Seeber, Martin Blume

**Affiliations:** Robert Koch Institute Berlin Germany; Robert Koch Institute Berlin Germany; Robert Koch Institute Berlin Germany; Robert Koch Institute Berlin Germany; Robert Koch Institute Berlin Germany; Robert Koch Institute Berlin Germany

The following is the authors’ response to the previous reviews

**Public Reviews:**

**Reviewer #1 (Public review):**

Summary:

The authors' goal was to advance the understanding of metabolic flux in the bradyzoite cyst form of the parasite *T. gondii*, since this is a major form of transmission of this ubiquitous parasite, but very little is understood about cyst metabolism and growth.

Nonetheless, this is an important advance in understanding and targeting bradyzoite growth.

Strengths:

The study used a newly developed technique for growing *T. gondii* cystic parasites in a human muscle-cell myotube format, which enables culturing and analysis of cysts. This enabled screening of a set of anti-parasitic compounds to identify those that inhibit growth in both vegetative (tachyzoite) forms and bradyzoites (cysts). Three of these compounds were used for comparative Metabolomic profiling to demonstrate differences in metabolism between the two cellular forms.

One of the compounds yielded a pattern consistent with targeting the mitochondrial bc1 complex, and suggest a role for this complex in metabolism in the bradyzoite form, an important advance in understanding this life stage.

Weaknesses:

Studies such as these provide important insights into the overall metabolic differences between different life stages, and they also underscore the challenge with interpreting individual patterns caused by metabolic inhibitors due to the systemic level of some of some targets, so that some observed effects are indirect consequences of the inhibitor action. While the authors make a compelling argument for focusing on the role of the bc1 complex, there are some inconsistencies in the some patterns that underscore the complexity of metabolic systems.

Thank you for reviewing the revised manuscript.

**Reviewer #2 (Public review):**

Summary:

A particular challenge in treating infections caused by the parasite *Toxoplasma gondii* is to target (and ultimately clear) the tissue cysts that persist for the lifetime of an infected individual. The study by Maus and colleagues leverages the development of a powerful in vitro culture system for the cyst-forming bradyzoite stage of Toxoplasma parasites to screen a compound library for candidate inhibitors of parasite proliferation and survival. They identify numerous inhibitors capable of inhibiting both the disease-causing tachyzoite and the cyst-forming bradyzoite stages of the parasite. To characterize the potential targets of some of these inhibitors, they undertake metabolomic analyses. The metabolic signatures from these analyses lead them to identify one compound (MMV1028806) that interferes with aspects of parasite mitochondrial metabolism. In the revised version of the manuscript, the authors present convincing evidence that MMV1028806 targets the mitochondrial electron transport (ETC) chain of the parasite (although they don't identify the actual target in the ETC). The revised manuscript also nicely addresses my other criticisms of the original version. Overall, the study presents an exciting approach for identifying and characterizing much-needed inhibitors for targeting tissue cysts in these parasites.

Strengths:

The study presents convincing proof-of-principle evidence that the myotube-based in vitro culture system for *T. gondii* bradyzoites can be used to screen compound libraries, enabling the identification of compounds that target the proliferation and/or survival of this stage of the parasite. The study also utilizes metabolomic approaches to characterize metabolic 'signatures' that provide clues to the potential targets of candidate inhibitors. In addition to insights into candidate bradyzoite inhibitors, the study also provides new insights into the physiological role of the mitochondrial electron transport chain of bradyzoites, and raises a host of interesting questions around the functional roles of mitochondria in this stage of the parasite.

Weaknesses:

In the revised manuscript, the authors have included additional oxygen consumption rate data that indicate that MMV1028806 targets the mitochondrial electron transport chain (ETC). These data are convincing. On line 481, the authors state that "treatments with ATQ, BPQ, MMV1028806, and antimycin A resulted in substantially reduced oxygen consumption levels relative to the DMSO control and suggest indeed a blockage of the mETC consistent with the inhibition of the bc1-complex." The OCR assay the authors use is still only an indirect measure of bc1 activity. Given that most OCR-inhibiting compounds in *T. gondii* are bc1 inhibitors, it is possible (and perhaps likely) that MMV1028806 is targeting this complex. However, the data cannot rule out that it is targeting another component of the ETC (or potentially even a TCA cycle enzyme). Without a direct test that MMV1028806 inhibits bc1 complex activity, the authors should be more cautious in their interpretation (e.g. by acknowledging the limitations of their conclusion, or acknowledging other possible targets). Similarly, the conclusion on line Line 622 that "... we confirmed the bc1-complex as a target" is overstating the findings. The phrasing on lines 683-695 is more appropriate: "... suggesting that it also targets complex III or a functionally linked site within the mitochondrial electron transport chain."

We are grateful for he thorough review of the updated manuscript and the identification the minor issues. We addressed all of them as detailed below. We also tempered our conclusions regarding the identification of the bc1-complex as a target in line 616:

“In addition to abundance data, Additionally, we confirmed the bc1-complex as a target by monitoring the incorporation of ^13^C and ^15^N stable isotopes from glucose and glutamine, respectively, into TCA cycle and pyrimidine biosynthesis intermediates suggest the bc1-complex as a target”

**Reviewer #3 (Public review):**

Summary:

The authors described an exciting 400-drug screening using a MMV pathogen box to select compounds that effectively affect the medically important Toxoplasma parasite bradyzoite stage. This work utilises a bradyzoites culture technique that was published recently by the same group. They focused on compounds that affected directly the mitochondria electron transport chain (mETC) bc1-complex and compared with other bc1 inhibitors described in the literature such as atovaquone and HDQs. They further provide metabolomics analysis of inhibited parasites which serves to provide support for the target and to characterise the outcome of the different inhibitors.

Strengths:

This work is important as, until now, there are no effective drugs that clear cysts during *T. gondii* infection. So, the discovery of new inhibitors that are effective against this parasite-stage in culture and thus have the potential to battle chronic infection is needed. The further metabolic characterization provides indirect target validation and highlight different metabolic outcome for different inhibitors. The latter forms the basis for new studies in the field to understand the mode of inhibition and mechanism of bc1-complex function in detail.

The authors focused in the function of one compound, MMV1028806, that is demonstrated to have a similar metabolic outcome to burvaquone. Furthermore, the authors evaluated the importance of ATP production in tachyzoite and bradyzoites stages and under atovaquone/HDQs drugs.

Thank you for reviewing the revised manuscript.

**Recommendations for the authors:**

**Reviewer #1 (Recommendations for the authors):**

Thanks for making appropriate updates. I believe it makes the report stronger. Just please double-check proof-reading in newly added text: for example "integration" is misspelled in Figure 4 legend (C, E).

Typos have been corrected throughout the manuscript.

**Reviewer #2 (Recommendations for the authors):**

I congratulate the authors on an excellent study. I have several minor comments for the authors to consider before publication.

Line 99. Schistosoma –

Corrected

Line 123. What was the pH of the bicarb-free RPMI medium?

Added “at pH 7.2”

Line 218 (and again on line 687). "RHku80" - are these just standard RH strain parasites? Or do the authors mean to imply that the ku80 gene has been knocked out in this line? If the latter, RH∆ku80 may be a better way to describe this line.

We harmonized all mentions of this strain to RH∆ku80.

Line 225. "Parasites were incubated in medium with one of the following treatments ..." How long were the parasites incubated in the different treatments before the plate was read? Was there any preincubation? I think not, but it would help to state this so the reader can appreciate that the effects of the compounds on OCR is likely an immediate (rather than a secondary) effect.

This is indeed a good suggestion. There was no pre-incubation and we added changed the text to: “Parasites were incubated in medium with one of the following treatments immediately before measurement: … “

Figure S2A. Check the spelling of Toxoplasmosis.

Done, we corrected this sentence.

Figure S2B. do you mean 'tachyzoidal' or 'tachyzocidal'? 'bradyzoidal' or 'bradyzocidal'?

We clarified the formulation of the legends for Fig S2.

Figure S2D. The "Tachyzoite lowest cytotoxicity" and "Bradyzoite lowest cytotoxicity" columns are, I think, depicting compound toxicity in host cells. Would it be clearer to rename these columns relative to the host cells being tested? e.g. "HFF/KD3 myotube lowest cytotoxicity"

Good suggestion and we changed the designation accordingly.

Line 369. "We found that tachyzocidal, bradyzocidal and dually active compounds possess a statistically significantly higher lipophilicity and this trend appeared more accentuated for bradyzocidal and dually active compounds." Significantly higher than what? Need to be clearer about the comparison being made: i.e. to non-active compounds.

You are correct and we corrected this sentence accordingly.

Line 500. "we attribute these changes to inhibition of host mitochondria (Fig. 5A)." The reason for referencing Figure 5A here isn't clear. Do the authors mean to point out that host mitochondrial membrane potential is affected by compound treatment? This could be stated more clearly.

We deleted the reference to Fig 5A. We did not systematically measure the effect of the inhibitors on the membrane potential of the host mitochondria. We also changed the sentence to emphasize the speculative nature of this assertion: “we attribute these changes to potential inhibitory effects on host mitochondria”.

Line 840. 'hurdling mechanisms'. The authors don't explain what they mean by this expression.

We truncated the figure title to: “Untargeted metabolomic analysis of bradyzoites treated with bc1-complex inhibitors shows an energy imbalance.”
